# “Redox Imaging” to Distinguish Cells with Different Proliferative Indexes: Superoxide, Hydroperoxides, and Their Ratio as Potential Biomarkers

**DOI:** 10.1155/2019/6373685

**Published:** 2019-04-08

**Authors:** Zhivko Zhelev, Ekaterina Georgieva, Dessislava Lazarova, Severina Semkova, Ichio Aoki, Maya Gulubova, Tatsuya Higashi, Rumiana Bakalova

**Affiliations:** ^1^Medical Faculty, Trakia University, 11 Armejska Str., Stara Zagora 6000, Bulgaria; ^2^Institute of Biophysics and Biomedical Engineering, Bulgarian Academy of Sciences, 21 Acad. G. Bonchev Str., Sofia 1113, Bulgaria; ^3^Medical Faculty, Sofia University, 1 Koziak Str., Sofia 1407, Bulgaria; ^4^Quantum-state Controlled MRI Group, Institute of Quantum Life Science, National Institutes for Quantum and Radiological Science and Technology (QST), 4-9-1 Anagawa, Chiba 263-8555, Japan; ^5^Functional and Molecular Imaging Team, Department of Molecular Imaging and Theranostics, National Institute of Radiological Science (NIRS), 4-9-1 Anagawa, Chiba 263-8555, Japan

## Abstract

The present study was directed to the development of EPR methodology for distinguishing cells with different proliferative activities, using “redox imaging.” Three nitroxide radicals were used as redox sensors: (a) mito-TEMPO—cell-penetrating and localized mainly in the mitochondria; (b) methoxy-TEMPO—cell-penetrating and randomly distributed between the cytoplasm and the intracellular organelles; and (c) carboxy-PROXYL—nonpenetrating in living cells and evenly distributed in the extracellular environment. The experiments were conducted on eleven cell lines with different proliferative activities and oxidative capacities, confirmed by conventional analytical tests. The data suggest that cancer cells and noncancer cells are characterized by a completely different redox status. This can be analyzed by EPR spectroscopy using mito-TEMPO and methoxy-TEMPO, but not carboxy-PROXYL. The correlation analysis shows that the EPR signal intensity of mito-TEMPO in cell suspensions is closely related to the superoxide level. The described methodology allows the detection of overproduction of superoxide in living cells and their identification based on the intracellular redox status. The experimental data provide evidences about the role of superoxide and hydroperoxides in cell proliferation and malignancy.

## 1. Introduction

Redox signaling is a key mechanism in maintaining cell homeostasis and normal functioning of the living organisms. Violations of this mechanism play a crucial role in the pathogenesis of many diseases: cancer, neurodegeneration, atherosclerosis, inflammation, diabetes, etc., whose common characteristic is the development of *oxidative stress* and impairment of redox balance in cells, tissues, and body fluids [1].

*Reactive oxygen species (ROS)* are the main inducers of oxidative stress. Their production can be accelerated by exogenous and/or endogenous factors [2, 3]. Some of the most popular exogenous inducers of ROS are radiation, heavy metals, and xenobiotics (including drugs, bacteria, viruses, and toxins). Endogenous inducers of ROS are predominantly mitochondria and enzyme complexes [NAD(P)H-dependent oxidases (NOX), cytochrome P450-dependent monooxygenases, xanthine oxidase, myeloperoxidase, and nitric oxide synthase (NOS)].

In the last decade, many researchers have confirmed that ROS are not just by-products of the mitochondria and enzyme complexes but important signal molecules that regulate many biochemical and physiological processes, from metabolism to immune response [4–7]. Some of the most attractive and widely analyzed species, found to be involved directly or indirectly in cell signaling, are superoxide (O_2_^·^-), hydrogen peroxide (Н_2_О_2_), nitric oxide (NO), and peroxynitrite (ONOO-). The pathogenic effects of ROS occur at over threshold concentrations. The endogenous *reducing equivalents* (e.g., antioxidant systems; thiol-containing proteins such as thioredoxin, peroxyderoxin, and glutaredoxin; and cofactors such as NADH and NADPH) are the main intracellular compounds to maintain ROS within physiological concentrations.

ROS and reducing equivalents are often described as “redox-active compounds,” and the balance between them as “redox status,” “redox state,” or “bioreduction capacity” of cells, tissues, and body fluids [8, 9]. Changes in their spatial and temporal distribution play a central role in pathogenesis [10]. Therefore, the redox status is considered important diagnostic marker and also a therapeutic target for all pathologies associated with a disturbance of cellular redox signaling.

In this context, the analysis of the redox status in cells, tissues, and body fluids is of particular importance and the perfect mythological approach should provide direct and noninvasive detection in vivo.

Significant progress has been made in the selective localized detection of many redox-active compounds (different types of ROS, various endogenous redox pairs, thiol-containing proteins, antioxidant enzymes, nonenzymatic antioxidants, etc.) [8, 9, 11, 12]. This progress is due to the development of new synthetic or genetically encoded redox-sensitive contrast substances, as well as to the improvement of the visualization techniques: fluorescent, chemiluminescent, magnetic resonance, nuclear, and ultrasonic.

At present, the efforts are focused on mapping the redox status of tissues and organs in intact organisms. This innovative approach is crucial for tracking the development of the diseases, accompanied by oxidative stress and impaired redox signaling, their prophylaxis and control over their therapy, predicting the effectiveness of therapy, and planning the therapeutic strategy—important factors in the personalized medicine.

There are many contrast substances that form detectable products reflecting the localization and level of a particular redox-active compound or group of compounds in the investigated biological object (Table 1S—see Supplementary Materials). The detection of most of them (e.g., fluorescent contrast agents) is feasible with high sensitivity and resolution in vitro but is very difficult to implement in vivo. In another group of contrasts (e.g., nuclear and ultrasound), it is possible to achieve in vivo detection with high sensitivity, but the resolution is low. Generally, nuclear contrast agents provide indirect information about the tissue redox status, based on its relationship to various biochemical and physiological processes (glucose or oxygen consumption, hypoxia, cell retention depending on the cytoplasmic redox potential, etc.). These contrasts are also radioactive, which adds a risk to the patient. It should be noted that using the methodological approaches listed above, the assessment of the redox status of the biological object is based on information, obtained only for one or several redox-active compounds, which may change simultaneously in different directions—one to increase and another to decrease or to remain on a steady-state level. Thus, the conclusions in different studies are often contradictory. The total redox status of cells can only be estimated by taking into account the sum of the redox status of the multiple intracellular redox pairs, which is currently impossible. Furthermore, the analysis of all intracellular redox pairs (reduced/oxidized states) is a time- and cost-consuming process.

We believe that these limitations could be overcome by developing a “perfect” redox sensor that should meet the following conditions:

- To penetrate in cells and through the blood-brain barrier (BBB), if possible
- To provide information about the equilibrium between the intracellular oxidizers and reducers, respectively, for the total redox status of cells and tissues, not only for the status of a certain redox pair (e.g., its oxidized or reducing form)
- To be nontoxic or low toxic in vivo
- To have a rapid excretion through the organism
- To be characterized by high contrast and to allow imaging with high resolution

Some of the most attractive redox-sensitive contrast substances are *cyclic nitroxide radicals*, which can be registered and analyzed in vitro and in vivo by various magnetic resonance techniques, such as electron paramagnetic resonance imaging (EPR), magnetic resonance imaging (MRI), and Overhauser-enhanced MRI (OMRI) [8, 12–15]. They allow an assessment of the redox status in cells, tissues, and body fluids.

**Figure 1:**
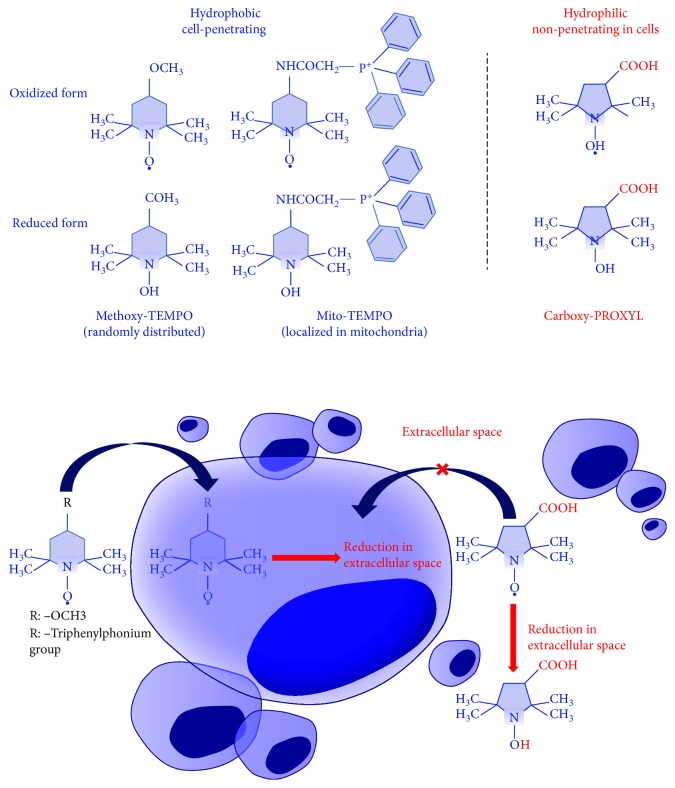
Structural formulas of nitroxide probes used in our study and potential mechanism of their redox transformations in living cells and tissues. Mito-TEMPO and methoxy-TEMPO, which penetrate the cells, allow the analysis of the intracellular redox capacity, while carboxy-PROXYL, which does not penetrate the cells, allows the analysis of the extracellular redox capacity (according to Soule et al. [13]).

Briefly, the paramagnetic nitroxide radical is involved in electron transfer reactions with various oxidizers and reducers, leading to the formation of diamagnetic intermediate products (hydroxylamine and oxoammonium). The rate constants of these reactions determine the dynamics of nitroxide-enhanced EPR/MRI contrast in living cells and body fluids (Figure 1S—see Supplementary Materials) [8, 9, 16–24]. It was found that nitroxide radical could be converted rapidly to the noncontrast hydroxylamine and/or oxoammonium by the following compounds: free ions of transition metals, hydroxyl and hydroperoxyl radicals, ubiquinols, NAD(P)H, ascorbate, etc. [12, 21, 22]. In turn, hydroxylamine and oxoammonium are superoxide dismutase (SOD) “mimetics” and could restore the nitroxide radical [17, 21–24]. The interaction of oxoammonium with superoxide occurs at pH < 4.5, whereas under physiological conditions (pH~7.4), the oxoammonium is reduced by NAD(P)H to hydroxylamine [12, 17]. The interaction of hydroxylamine with superoxide occurs at approximately pH 7.4 with the release of hydrogen peroxide and restoration of the radical nitroxide form [12, 17, 24]. It is generally accepted that in living cells and tissues, nitroxide exists primarily in two forms: as a radical and as a hydroxylamine. Various reducers and oxidizers are involved (directly or indirectly via oxoammonium) in the formation of hydroxylamine, but only the interaction of hydroxylamine with superoxide is the process that restores the nitroxide radical and its MRI/EPR contrast. Thus, the dynamics of the EPR/MRI signal of cell-penetrating nitroxide radicals in cell suspensions follows the intracellular redox status and could serve as a marker of oxidative stress, accompanied by overproduction of superoxide.

One of the earliest methods for the detection of superoxide using EPR spectroscopy was spin trapping with 5,5-dimethyl-1-pyrroline-N-oxide (DMPO) [25]. It is important to distinguish between spin traps and spin probes. Spin traps form a covalent bond with ROS, while spin probes (such as cyclic nitroxide radicals) are oxidized by ROS without binding [12]. DMPO and other nitrone spin traps are very useful in studies of isolated biomacromolecules (e.g., enzymes) and in pure chemical systems. However, they react with superoxide at very slow rate constants and it is difficult to use them for the detection of superoxide in biological systems due to competition with SOD and intracellular reducers (such as ascorbate) [12].

Another nitrone spin trap—5-diethoxyphosphoryl-5-methyl-1-pyrroline N-oxide (DEPMPO)—has been conjugated with the triphenylphosphonium group, which allows a selective penetration into the mitochondria and interaction with mitochondrial superoxide [26]. Unfortunately, the use of this spin trap in living cells is limited by its slow interaction with superoxide, potential toxicity, and nonspecific effects [12, 27, 28]. Mito-DEPMPO has to be used at high concentrations (50 mM), which may cause inhibition of mitochondrial respiration due to the accumulation of large amounts of lipophilic cations in the mitochondrial matrix and disruption of the mitochondrial potential [12, 29].

Dikalov et al. have reported that cyclic hydroxylamines can be used for the measurement of superoxide in cultured cells and tissues and in vivo [30–32]. These diamagnetic molecules are oxidized by superoxide and other ROS to form paramagnetic EPR-detectable stable nitroxide radicals in cell cultures [12, 23, 30–32]. Cyclic hydroxylamines react with superoxide much more rapidly than nitrone spin traps, which enhances the efficiency for the detection of intracellular superoxide [32].

The cyclic nitroxide radicals and their diamagnetic hydroxylamines can be used at relatively low concentrations (0.05-1 mM), minimizing toxic side effects in biological systems. Nitroxide radicals and their hydroxylamines possess also antioxidant properties [14], which increases their potential for in vivo application.

The present study is aimed at investigating the possibilities of using cyclic nitroxide radicals (mito-TEMPO, methoxy-TEMPO, and carboxy-PROXYL) as contrast probes for analyzing the intracellular redox status under oxidative stress induced by endogenous or exogenous factors. The experiments were conducted on isolated cell lines with different proliferative activities and oxidative capacities. The efforts were directed to the development of EPR methodology for distinguishing cells with different proliferative activities, based on their different redox status and using “redox imaging.” The experimental data provide evidences about the role of superoxide and hydroperoxides in cell proliferation and malignancy.

## 2. Materials and Methods

### 2.1. Chemical Reagents

Mito-TEMPO [((2-(2,2,6,6-tetramethylpiperidin-1-oxyl-4-ylamino)-2-oxoethyl)triphenylphosphonium chloride)], methoxy-TEMPO (4-methoxy-2,2,6,6-tetramethyl-1-piperidinyloxy), and carboxy-PROXYL (3-carboxy-2,2,5,5-tetramethyl-1-pyrrolidinyloxy) were purchased from Sigma-Aldrich. All the reagents used in the experiments were “HPLC-grade” and “TOF-MASS grade.” In all experiments, we used ultrapure deionized water (Milli-Q for TOF-MASS).

### 2.2. Cells

Two types of human cell lines: noncancer/normal (FHC, HaCaT, HUVEC, and normal lymphocytes) and cancer (HT29, Colon26, Neuro2a, OSC19, U87, Jurkat, K562, and Raji), were used. Cancer cell lines were isolated from patients with Burkitt lymphoma (Raji), chronic myeloid leukemia (K-562), acute lymphoblastic leukemia (Jurkat), colorectal carcinoma (Colon26), glioma (U87), and osteosarcoma (OSC19). The cell lines were purchased from the RIKEN Bioresource Center (Saitama, Japan), Thermo Fisher Scientific (Tokyo, Japan), and American Type Culture Collection (ATCC; Manassas, VA, US).

The nonadhesive cell lines were cultured in RPMI-1640, containing 10% of heat-inactivated fetal bovine serum (FBS) and antibiotics (100 *μ*g/mL of streptomycin and 100 U/mL of penicillin). The adhesive cancer cell lines and HaCaT were cultured in Dulbecco's modified Eagle's medium (DMEM), HUVEC in endothelial cell growth medium (ECGM), and FHC in DMEM-F12, supplemented with growth factors. All mediums were supplemented with 10% FBS.

Normal lymphocytes were isolated from the heparinized blood of clinically healthy donors by using Lymphosepar-I (Immuno-Biological Laboratories, Takasaki, Japan), followed by five to seven times washing with phosphate-buffered saline (PBS, 10 mM, рН 7.4), before replacement in cell cultured medium (RPMI-1640). The multistep washing was very important due to necessity to remove completely the free iron from the suspension, which is a result of eventual hemolysis of erythrocytes during the procedure. The transition metal ions can induce Fenton's type reactions and compromise the EPR measurements and data analysis. All experiments on normal lymphocytes were carried out in a 10-day period after their isolation. Then, the isolation procedure was repeated using fresh heparinized blood.

All cell lines were grown in an incubator at 37°C and a humidified atmosphere, saturated with 5% CO_2_.

Twenty-four hours before the experiment, the cells were replaced in a fresh medium without antibiotics. To remove the adhesive cells from the plates, we used a trypsin-EDTA solution (0.5% of trypsin, 0.2% of EDTA) and subsequent washing with PBS. During the culturing and experiments, the cells were sedimented by centrifugation (1000×g/10 min for nonadhesive or 800×g/5 min for adhesive). The cells were washed three times with 10 mM PBS (pH 7.4), if necessary (depending on biochemical analysis).

### 2.3. Cell Proliferation and Cytotoxicity Assay

Cell viability and proliferation were analyzed by trypan blue staining and automated counting, using Countess™ Automated Cell Counter (Invitrogen, Oregon, USA).

Briefly, 10 *μ*L of trypan blue (0.4%) was added to 10 *μ*L of cell suspension, incubated for 30 s, and 10 *μ*L of the cell suspension was placed in a Countess® (Invitrogen) glass chamber. The number of live and dead cells in the suspension was counted automatically. The linear range to operate with the automated cell counter was 1 × 10^4^ − 5 × 10^6^ cells/mL, and the optimal cell size was in the range of 5-60 *μ*m.

### 2.4. EPR Spectroscopy

EPR experiments were performed on an X-Band spectrometer (JEOL, Tokyo, Japan) with standard cavity, at the following parameters: microwave frequency: 9.4 GHz; field strength = 336 mT; field modulation frequency = 100 kHz; field modulation amplitude = 0.063 mT; microwave power: 2.0 mW; time constant = 0.01 s; sweep width = 10 mT; scan time s; and a number of scans: 1. The EPR spectra were integrated, and the data were calculated as a percentage from the control (0.1 mM of nitroxide dissolved in cell-free medium).

In all EPR experiments, cells were collected by centrifugation and resuspended in the respective medium without supplements.

### 2.5. Dihydroethidium (DHE) Assay

DHE is a cell-penetrating fluorogenic probe, interacting predominantly with superoxide [12, 33]. It allows distinguishing between superoxide and hydrogen peroxide and analyzing the level of intracellular superoxide.

Briefly, DHE was dissolved in DMSO to 65 mM stock solution (kept at -40°C), which was diluted with PBS to prepare 50 *μ*M of DHE working solution in the day of experiment. Ten *μ*L of DHA (50 *μ*M) was added to 1 mL of cell suspension (1 × 10^6^ cells/mL). The samples were incubated within 15 min at room temperature, washed three times with PBS using centrifugation, and finally resuspended in 500 *μ*L of PBS. The fluorescence intensity was detected immediately at *λ*_ex_ = 518 nm and *λ*_em_ = 605 nm, using a microplate reader (TECAN Infinite® M1000, Austria).

Three parallels were prepared for each sample.

### 2.6. Dihydrodichlorofluorescein (DCF) Assay

2,7-Dichlorodihydrofluorescein-diacetat (DCFH-DA) is a cell-penetrating fluorogenic probe, interacting predominantly with hydrogen peroxide [12]. It allows analyzing the level of intracellular hydroperoxides, using an OxiSelect™ In vitro ROS Assay kit (Green Fluorescence) (Cell Biolabs Inc., US).

Briefly, 100 *μ*L of aliquots of cells (1 × 10^6^ cells/mL) was placed in 96-well plates. Ten *μ*L of DCFH-DA was added to each well. The samples were incubated within 60 min at room temperature, washed three times with PBS using centrifugation, and finally resuspended in 100 *μ*L of PBS. The fluorescence intensity was detected immediately at *λ*_ex_ = 480 nm and *λ*_em_ = 530 nm, using a microplate reader (TECAN Infinite® M1000, Austria). DCF was used as a standard.

Three parallels were prepared for each sample.

### 2.7. Superoxide Dismutase (SOD) Activity Assay

Superoxide dismutase activity was analyzed, using a Superoxide Dismutase Activity Assay kit (Abcam, Tokyo, Japan). Cell lysates were prepared in the absence of proteolytic enzymes, as described in the manufacturer's instruction. A xanthine/xanthine oxidase (X/XO) system is used to generate superoxide in the cell lysate. The superoxide interacts with WST-1 electron coupling reagent, which results in a production of a formazan product with absorbance maximum at 450 nm. SOD converts superoxide to hydrogen peroxide, yielding less colorimetric signal at 450 nm.

Briefly, 20 *μ*L of cell lysate (obtained from 3 × 10^6^ cells/mL) were placed in 96-well plates. Two hundred *μ*L of WST-1 working solution and 20 *μ*L of enzyme working solution were added to each well. The samples were incubated within 20 min at 37°C. The absorbance at 450 nm was detected, using a microplate reader (TECAN Infinite® M1000, Austria).

Three parallels were prepared for each sample. The data were calculated as a percentage of inhibition of X/XO reaction by the SOD in the cell lysates.

### 2.8. Catalase Activity Assay

Catalase activity was analyzed, using an OxiSelect™ Catalase Activity Assay kit (Cell Biolabs Inc., US). Cell lysates were prepared in the absence of proteolytic enzymes, as described in the manufacturer's instruction. The catalase-containing lysate is incubated with a known amount of hydrogen peroxide. The reaction proceeds for exactly 1 min. Then, catalase is inhibited by sodium azide. The remaining hydrogen peroxide, following a fixed incubation period, is then determined by the oxidative coupling reaction of 4-aminophenazone (AAP) and 3,5-dichloro-2-hydroxy-benzenesulfonic acid (DHBS) in the presence of horseradish peroxidase (HRP). The resulting quinoneimine dye is measured at 520 nm, which correlates to the amount of hydrogen peroxide remaining in the reaction mixture. Catalase with known activity is used as a standard.

Briefly, 20 *μ*L of cell lysate (obtained from 3 × 10^6^ cells/mL) was placed in 96-well plates. Fifty *μ*L of hydrogen peroxide (12 mM) was added to each well and incubated for 1 min at room temperature. Catalase activity was stopped by the addition of 50 *μ*L of catalase quencher (sodium azide). Five *μ*L aliquots were transferred to new wells. Two hundred and fifty *μ*L of chromogen solution was added to each well and incubated for 60 min at room temperature with mixing.

The control samples contained catalase standard instead of cell lysate. The absorbance at 520 nm was detected, using a microplate reader (TECAN Infinite® M1000, Austria).

Three parallels were prepared for each sample. The data were calculated as a percentage of inhibition of HRP reaction by the catalase in the cell lysates.

### 2.9. Glutathione Peroxidase (GSH-Px) Activity Assay

Glutathione peroxidase activity was analyzed, using a Glutathione Peroxidase Assay kit (Abcam, Tokyo, Japan). Cell lysates were prepared in the absence of proteolytic enzymes, as described in the manufacturer's instruction. Glutathione peroxidase reduces cumene hydroperoxide while oxidizing GSH to GSSG. The generated GSSG is reduced to GSH by glutathione reductase with consumption of NADPH. The decrease of NADPH is detected at 340 nm, which is proportional to glutathione peroxidase activity.

Briefly, 50 *μ*L of cell lysate (obtained from 3 × 10^6^ cells/mL) was incubated with 40 *μ*L of reaction mixture (40 mM of NADPH solution, glutathione reductase and GSH solution) within 15 min at room temperature to deplete all GSSG in the samples. Then, 10 *μ*L of cumene hydroperoxide was added to start the glutathione peroxidase reaction. The absorbance at 340 nm was detected within 10 min at room temperature, using a microplate reader (TECAN Infinite® M1000, Austria).

Three parallels were prepared for each sample. The data were calculated as “nmol NADPH consumed for 10 min” in the cell samples, compared to the control. Control sample contained all ingredients instead of cell lysate.

### 2.10. Total Antioxidant Capacity (TAC) Assay

The TAC assay was performed using an OxiSelect™ Total Antioxidant Capacity (TAC) Assay kit (Cell Biolabs Inc., US). The method is based on the reduction of Cu^2+^ to Cu^+^ by antioxidants and other reducing equivalents in the biological sample. Cu^+^ interacts with a chromophore to obtain a color product with an absorption maximum at 490 nm. The value of the absorption is proportional to the total antioxidant, respectively, reducing capacity of the biological object.

Briefly, cell lysates were prepared as it was described in the manufacturer's instruction. All lysates were adjusted to the same protein concentration. Aliquots of the cell lysates were placed in а 96-well plate. Each cell lysate was incubated with a copper ion reagent and chromophore as it was described in the instruction. The absorption of the product at 490 nm was detected by a microplate reader (Tecan Infinite F200 PRO, Austria). Three independent experiments were performed for each sample, with two parallel sample measurements for each experiment.

The total antioxidant capacity of the samples was determined by a calibration curve using uric acid as a standard. The results are presented as a “total antioxidant capacity (TAC),” which is equivalent to a “total reducing capacity” in “mM uric acid equivalents.” One mM of uric acid corresponds to 2189 *μ*M of Cu^2+^-reducing equivalents.

### 2.11. Glucose Consumption

Cells (3 × 10^5^ per well) were plated in 96-well plates, in a fresh medium for 24 h in a humidified atmosphere (5% CO_2_, 37°C). Then, the cells were counted and sedimented by centrifugation. The supernatant was analyzed for glucose concentration using a YSI glucometer (USA). Glucose consumption was determined by subtracting glucose concentration in the supernatant at 24 h point from the initial 0 h point. The data were normalized to the number of cells at each time point.

### 2.12. Statistical Analysis

All results are expressed as the means ± standard deviation (SD). Comparisons between the groups were performed using Student's *t*-test. A value of *p* < 0.05 was considered significant.

## 3. Results and Discussion

### 3.1. Dynamics of the EPR Signal of Nitroxide Radicals in Leukemic and Normal Lymphocytes

Three nitroxide radicals with different hydrophobicity and localization in living cells were used: (a) mito-TEMPO—strongly hydrophobic, penetrating through the cell membrane and localizing predominantly in the mitochondria; (b) methoxy-TEMPO—hydrophobic, penetrating through the cell membrane and randomly distributed between the cytoplasm and intracellular organelles; and (c) carboxy-PROXYL—hydrophilic, nonpenetrating in living cells and evenly distributed in the extracellular environment (Figure 1).

Initially, we analyzed the dynamics of the EPR signal of mito-TEMPO in leukemic and normal lymphocytes at different incubation times and different concentration ratios of cells/nitroxide. The nitroxide concentration was fixed (0.1 mM) in the optimal range for EPR measurements. The number of cells in the suspension varied from 1 × 10^6^ cells/mL to 7 × 10^6^ cells/mL.

**Figure 2:**
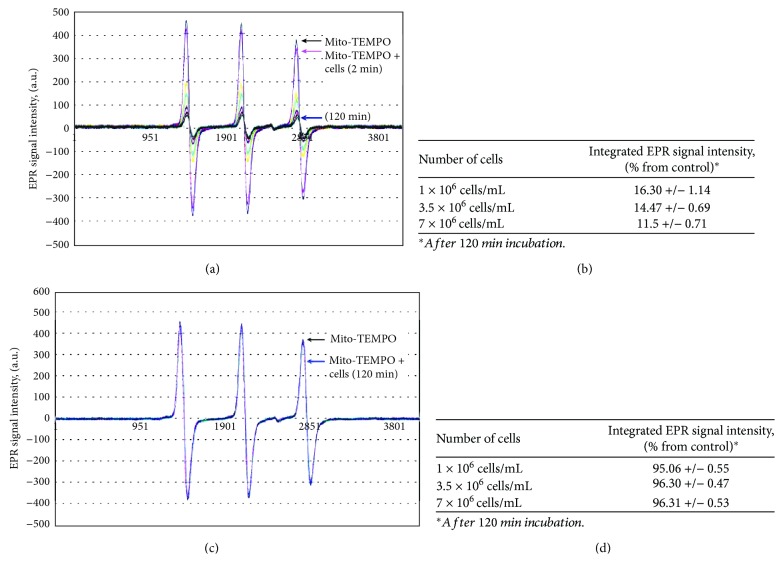
Representative EPR spectra of mito-TEMPO (0.1 mM) before and after incubation with cells (1 × 10^6^ cells/mL) at different time intervals. (a) Normal lymphocytes and (c) leukemic lymphocytes. Tables indicate the EPR signal intensity of mito-TEMPO, incubated with different numbers of cells for 2 hours, in a humidified atmosphere. (b) Normal lymphocytes and (d) leukemic lymphocytes. Control: mito-TEMPO in cultured (cell-free) medium. The data are the means ± SD from six independent experiments. The cell viability did not change at 2-hour incubation and was ~92-95% (for normal lymphocytes) or 96-99% (for leukemic lymphocytes).

Figure 2(a) represents EPR spectra of mito-TEMPO before and after incubation with normal lymphocytes. The signal intensity significantly decreased during incubation—about 85% of the control within 2 hours. Increasing the number of cells in the suspension was accompanied by a stronger EPR signal decay (Figure 2(b)). However, the difference between 1 × 10^6^ cells/mL and 7 × 10^6^ cells/mL was relatively small, which suggests that 1 × 10^6^ cells/mL are sufficient to convert almost the entire amount of nitroxide radical to its hydroxylamine form within 2 hours of incubation, at the selected experimental conditions. The data demonstrate that normal lymphocytes can convert the contrast radical form of mito-TEMPO into its noncontrast hydroxylamine form, which is evidence of their high reducing capacity.

Completely different EPR signal dynamics was detected before and after incubation of mito-TEMPO with leukemic lymphocytes (Jurkat) within 2 hours (Figure 2(c)). The signal intensity decreased very slightly during incubation—about 5% of the control. Increasing the number of cells in the suspension did not affect the level of EPR signal decay within 2 hours (Figure 2(d)). There are two possibilities: (a) mito-TEMPO is not converted into hydroxylamine due to the poor cell reducing ability of Jurkat, (b) or it is converted to hydroxylamine by the intracellular reducers and then converted back to nitroxide radical by the intracellular superoxide. The reduction of mito-TEMPO and the oxidation of mito-TEMPOH in the cells are competitive processes that run fast and simultaneously. The fact that the EPR signal slightly decreases in Jurkat (~5%) indicates that a small portion of the nitroxide is in a reduced form. In both options mentioned above, the data in Figure 2 show that leukemic lymphocytes possess higher oxidative capacity against mito-TEMPO than normal lymphocytes. Therefore, mito-TEMPO is a suitable contrast probe to distinguish normal and leukemic lymphocytes, based on their different redox status.

**Figure 3:**
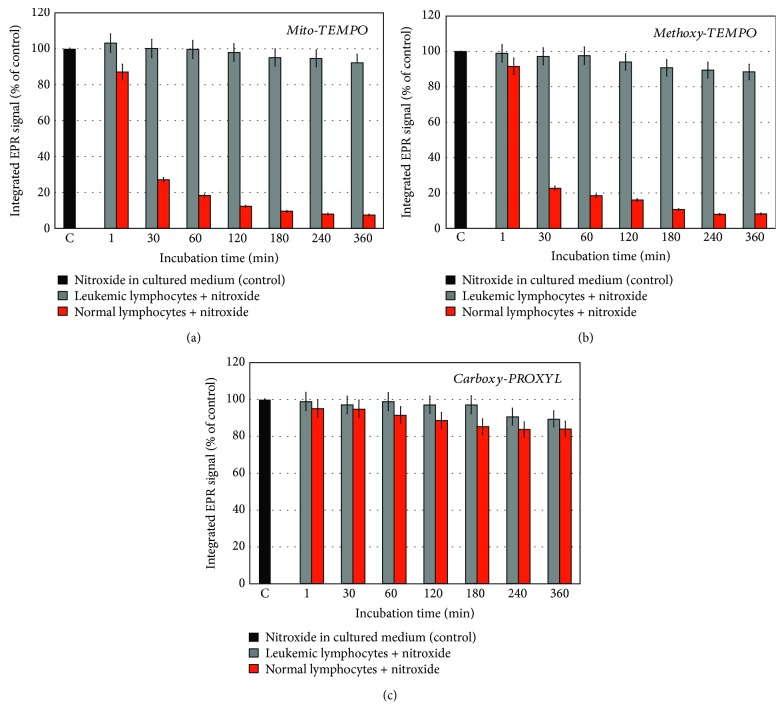
Dynamics of the EPR signal of nitroxide radical (0.1 mM) in leukemic lymphocytes (Jurkat) and normal lymphocytes (1 × 10^6^ cells/mL) during 6-hour incubation: (a) mito-TEMPO, (b) methoxy-TEMPO, and (c) carboxy-PROXYL. The data are the means ± SD from three independent experiments. The cell viability did not change at 6-hour incubation and was ~92-95% (for normal lymphocytes) or 96-99% (for leukemic lymphocytes).

We compared the dynamics of the EPR signal of three nitroxide radicals with different intracellular localization, during 6-hour incubation with normal and leukemic lymphocytes (Figure 3). Mito-TEMPO and methoxy-TEMPO were characterized by the same kinetics of the EPR signal in both cell suspensions (Figures 3(a) and 3(b)). The EPR signal of mito-TEMPO and methoxy-TEMPO significantly decreased after 6-hour incubation with normal lymphocytes (~90-95%) but slightly decreased after incubation with leukemic lymphocytes (~5-10%). In contrast, the EPR signal of carboxy-PROXYL followed the same kinetics in both cell lines (Figure 3(c)). The signal slightly decreased after 6-hour incubation—about 15% in normal lymphocytes and ~10% in leukemic lymphocytes. It is well known that carboxy-PROXYL has a difficult and slow penetration into the live cells due to its hydrophilicity [13, 34, 35]. Therefore, this nitroxide practically has no access to the intracellular reducers and oxidizers, at least for 6-hour incubation.

The data, obtained in this part of the study, suggest that normal and leukemic lymphocytes are characterized by a completely different redox status that can be analyzed by using EPR spectroscopy and cell-penetrating nitroxides, mito-TEMPO and methoxy-TEMPO, as redox sensors. Carboxy-PROXYL is not suitable for this purpose. The penetration of carboxy-PROXYL into the cells is a very slow process, which impedes its access to the intracellular redox-active compounds.

It is known that cancer cells, in particular leukemic lymphocytes, have a higher oxidative capacity than normal lymphocytes, despite the high level of endogenous reducers (such as NADH, NADPH, and antioxidants) as a result of their adaptation in response to prolonged oxidative stress [4, 5, 36–41]. The high levels of reducers in leukemic lymphocytes would lead to conversion of nitroxide radical to hydroxylamine and loss of EPR contrast. However, the high level of superoxide counteracts this process and would lead to the recovery of the nitroxide radical and EPR contrast. Normal lymphocytes are characterized by low basal levels of superoxide and higher levels of reducing equivalents [37, 40]. Most likely, this is the reason for the fast reduction of mito-TEMPO and methoxy-TEMPO in these cells.

### 3.2. Dynamics of the EPR Signal of Mito-TEMPO in Cells of Different Origins and Proliferative Activities: Correlation with the Levels of Intracellular Superoxide and Hydrogen Peroxide

In this stage of the study, we used (a) nonproliferating normal lymphocytes, (b) slowly proliferating noncancer cell lines—human umbilical vein endothelial cells (HUVEC) and human keratinocytes (HaCaT), and (c) rapidly proliferating cancer cell lines—Jurkat, K562, Raji, Colon26, U87, and OSC19.

**Figure 4:**
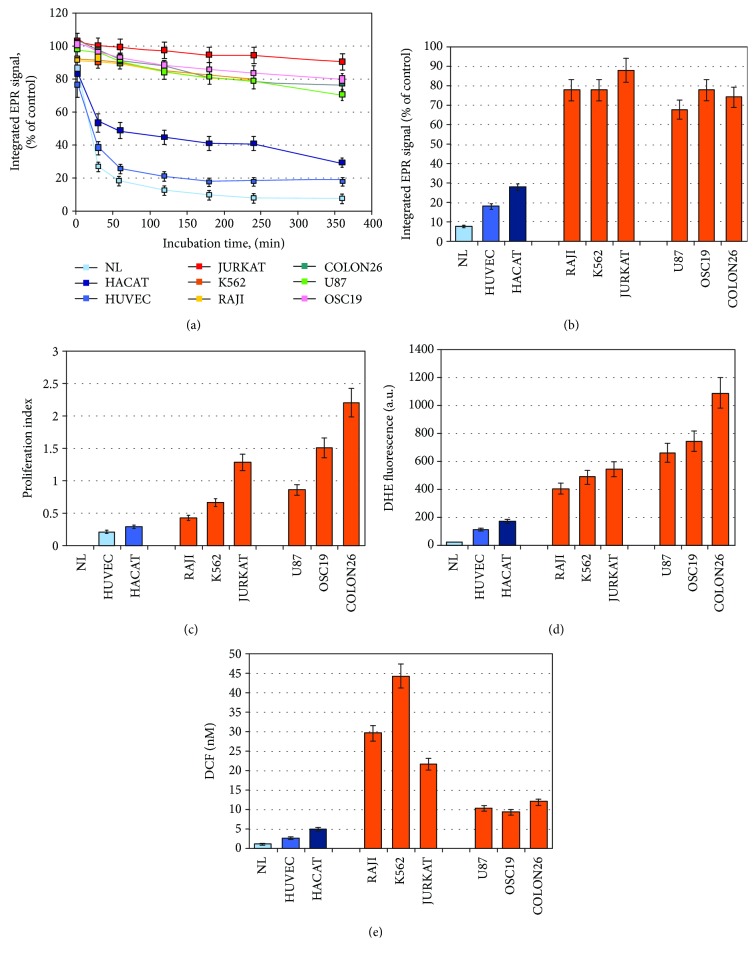
(a) Dynamics of the EPR signal of mito-TEMPO (0.1 mM) in cells with different proliferative indexes (1 × 10^6^ cells/mL) within 6-hour incubation. Control: mito-TEMPO in cultured medium. Mito-TEMPO did not affect cell viability at the selected experimental conditions. NL: normal lymphocytes. (b) Integrated EPR signal of mito-TEMPO in cell suspensions after 6-hour incubation. (c) Proliferative index of cells. (d) Basic intracellular levels of superoxide, analyzed by a DHE test. (e) Basic intracellular levels of hydroperoxides, analyzed by a DCF test. The data in (d) and (e) were normalized to 1 × 10^6^ cells/mL. In (a), (b), (c), and (d), the data are means ± SD from six independent experiments. In (e), the data are means ± SD from nine independent experiments.

In normal lymphocytes and slowly proliferating cells, we observed a rapid and significant EPR signal decay within 6-hour incubation: ~90% in normal lymphocytes, ~80% in HUVEC, and ~70% in HaCaT, compared to the control (mito-TEMPO in cell-free medium) (Figure 4(a)). In all cancer cell lines, a very slow decrease of the EPR signal was detected—up to 10-25% of the control level after 6-hour incubation.

The proliferative activity of the cells increased in the following order: normal lymphocytes < HUVEC < HaCaT < cancer cells (Figure 4(c)). The intensity of the EPR signal of mito-TEMPO in cell suspensions after 6-hour incubation increased in the same order (Figure 4(b)).

The next goal was to determine the level of oxidative stress in each cell line using two conventional methods: (a) DHE-assay, which is specific for detection of intracellular superoxide, and (b) DCF-assay, which is specific for the detection of intracellular hydroperoxides. It was found that the level of superoxide in cell suspensions increased in the following order: normal lymphocytes < HUVEC < HaCaT < cancer cells (Figure 4(d)). The levels of hydroperoxides varied widely, with no relation to their proliferative activity (Figure 4(e)). The highest levels of hydroperoxides were detected in Jurkat, K562, and Raji, but in U87, OSC19, and Colon26, the levels were commensurable with those detected in nonproliferating or slowly proliferating cells.

**Figure 5:**
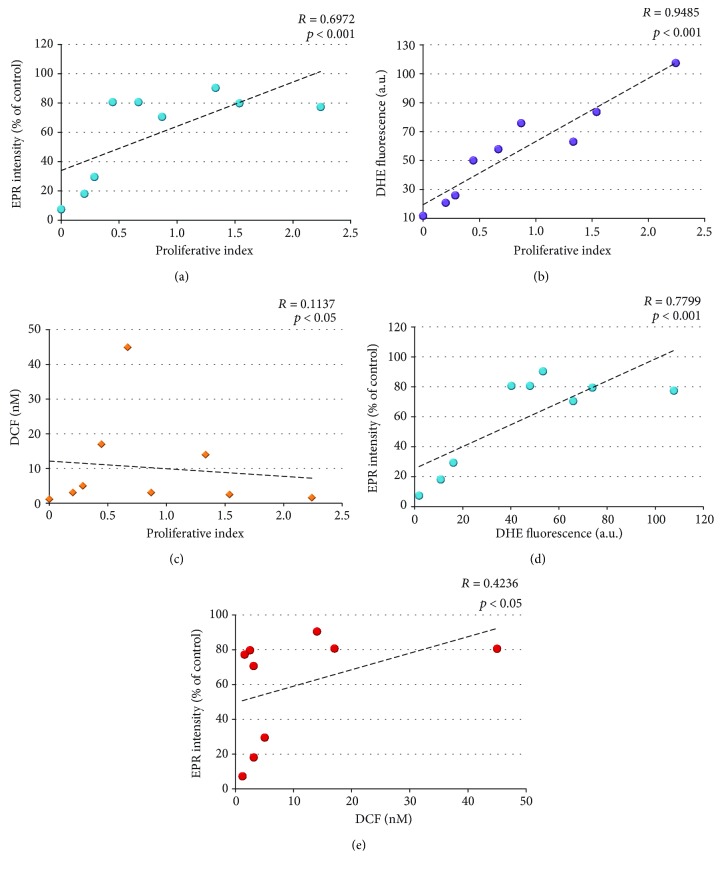
Correlation between the proliferative index, cellular redox status (analyzed by EPR spectroscopy), intracellular superoxide (analyzed by a DHE test), intracellular hydroperoxides (analyzed by a DCF test). *R*: correlation coefficients.

We found a good positive correlation between the EPR signal of mito-TEMPO and cellular proliferative index (*R* = 0.6972; *p* < 0.001) (Figure 5(a)), as well as between the EPR signal and superoxide level in the cells (*R* = 0.7788; *p* < 0.001) (Figure 5(d)). A very good positive correlation was also found between the superoxide level (analyzed by DHE) and proliferative index (*R* = 0.9485; *p* < 0.001) (Figure 5(b)). The correlations between the EPR signal and hydroperoxides level, as well as between the proliferative index and hydroperoxides level, were poor and/or nonsignificant (Figures 5(c) and 5(e)). There was no correlation between the levels of superoxide and hydroperoxides, analyzed by DHE and DCF (*R* = 0.0078; *p* > 0.05).

**Figure 6:**
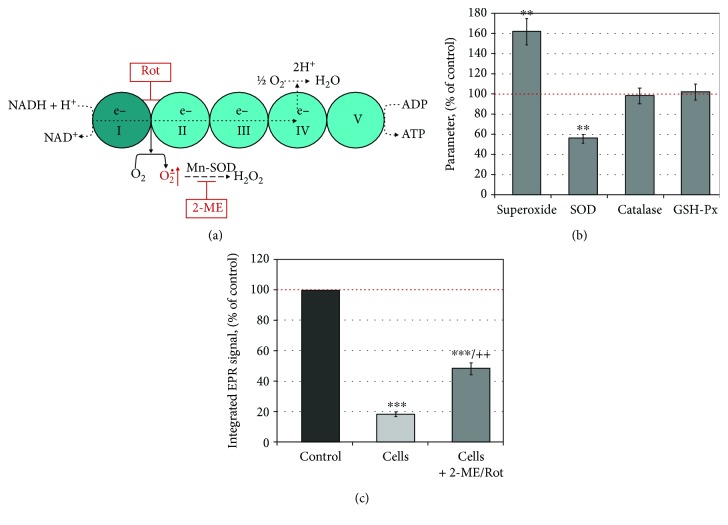
(a) Biochemical strategy to enhance superoxide accumulation in cells by inhibition of mitochondrial electron transport chain and mitochondrial superoxide dismutase (SOD2) (according to Pelicano et al. [44]). (b) Level of superoxide and activities of antioxidant enzymes in cells, treated with 2-ME/Rot. Experimental conditions: cells (normal lymphocytes; 1 × 10^6^ cells/mL) were preincubated in the absence or presence of 600 nM 2-ME and 500 nM Rot within 6 hours in a humidified atmosphere (5% CO_2_, 37°C). Control sample contained untreated cells. The superoxide level was measured by DHE assay, and the activities of antioxidant enzymes were measured as described in Materials and Methods. (c) EPR signal intensity of mito-TEMPO in untreated and 2-ME/Rot-treated cells. Experimental conditions: cells (1 × 10^6^ cells/mL) were preincubated in the absence or presence of 600 nM 2-ME and 500 nM Rot within 6 hours in a humidified atmosphere (5% CO_2_, 37°C). Mito-TEMPO (0.1 mM) was added to the cell suspensions, and the incubation was continued for 1 hour at the same conditions. Aliquots of the cell suspensions were collected and subjected to EPR analysis. Control sample contained mito-TEMPO (0.1 mM) in cultured (cell-free) medium. In (b) and (c), the data are the means ± SD from three and four independent experiments, respectively. ^∗∗^ *p* < 0.01 and ^∗∗∗^ *p* < 0.001 versus control; ++*p* < 0.01 versus cells only. Dotted red lines show the control levels. ME: 2-methoxyestradiol; Rot: rotenone; SOD: superoxide dismutase; GSH-Px: glutathione peroxidase.

The data suggest that the EPR intensity of mito-TEMPO correlates with the proliferative index and the level of oxidative stress, analyzed by the intracellular superoxide, but not by the intracellular hydroperoxides. Our previous studies on cell-free systems indicated that superoxide, but not hydrogen peroxide, is responsible for the recovery of the EPR contrast of nitroxide radical after its reduction to hydroxylamine (Figures 3S–6S—see Supplementary Materials) [42, 43]. Therefore, the described EPR methodology allows an assessment of the balance between the intracellular reducing equivalents and oxidizers and is a useful sensing platform for the detection of overproduction of superoxide in cell suspensions.

To clarify the role of intracellular superoxide in the dynamics of the EPR signal of mito-TEMPO, we used cells treated with rotenone (Rot) and 2-methoxyestradiol (2-ME). Rotenone is an inhibitor of the mitochondrial complex-I, and 2-methoxyestradiol is an inhibitor of Mn-SOD (Figure 6(a)) [44]. It has been found that the combination 2-ME/Rot causes an accumulation of high amounts of superoxide in the cells and strongly minimizes the production of hydrogen peroxide [42–44].

Normal lymphocytes were incubated with 2-ME/Rot within 6 hours in a humidified atmosphere. Several parameters were analyzed: level of superoxide, activities of antioxidant enzymes, and dynamics of the EPR signal of mito-TEMPO in 2-ME/Rot-treated and untreated cell suspensions (Figure 6(b)). SOD activity decreased about 45%, whereas the activities of catalase and glutathione peroxidase did not change significantly at the selected experimental conditions. Superoxide level increased markedly in 2-ME/Rot-treated cells—about 60% compared to the untreated cells. EPR signal intensity was about 2 times higher in 2-ME/Rot-treated cells compared to the untreated cells. These data demonstrate a lower rate of reduction of mito-TEMPO in 2-ME/Rot-treated cells, respectively, a higher oxidative capacity of these cells compared to the untreated. Overproduction of superoxide in the system indicates its role in the enhancement of EPR contrast. We also found on normal lymphocytes that the EPR signal decay is slower when increasing the concentration of 2-ME/Rot [43].

### 3.3. Dynamics of the EPR Signal of Nitroxide Radical in Cells of the Same Origin and Different Proliferative Activities: Correlation with the Levels of Intracellular Superoxide, Hydrogen Peroxide, and Antioxidant Enzymes

To elucidate the role of superoxides and hydroperoxides in the dynamics of the EPR signal of mito-TEMPO, we compared the redox status of normal and cancer cell lines of the same origin, but in different stages of differentiation and different SOD activities: FHC, HT29, and Colon26. All cell lines were epithelial, derived from colon mucosa: FHC—normal cells, HT29—stage II cancer cells, and Colon26—stage IV cancer cells.

**Figure 7:**
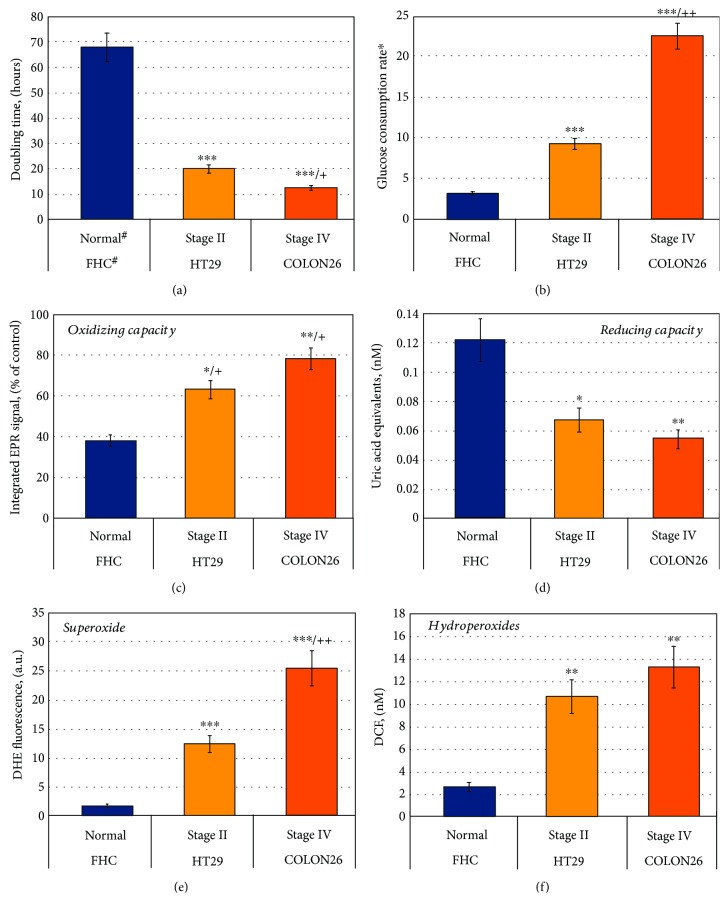
(a) Doubling time of normal (FHC) and cancer (HT29, Colon26) colon epithelial cells. ^∗^The doubling time of FHC was obtained in DMEM-F12 medium, supplemented with growth factors. (b) Glucose consumption in cells (^∗^ *μ*mol/1 × 10^6^ cells/24 hours). (c) EPR signal intensity of mito-TEMPO (0.1 mM) in cell suspensions (3 × 10^6^ cells/mL) within 6-hour incubation at 37°C. Control: mito-TEMPO (0.1 mM) in cultured medium. Mito-TEMPO did not affect cell viability at the selected experimental conditions. (d) Basal intracellular levels of antioxidants and reducing equivalents, analyzed by OxiSelect™ Total Antioxidant Capacity Assay. (e) Basal intracellular levels of superoxide, analyzed by a DHE test. (f) Basal intracellular levels of hydroperoxides, analyzed by a DCF test. The data in (c), (d), (e), and (f) were normalized to 1 × 10^6^ cells/mL. All data are the means ± SD from three independent experiments. ^∗^ *p* < 0.05, ^∗∗^ *p* < 0.01, and ^∗∗∗^ *p* < 0.001 versus FHC; +*p* < 0.05 and ++*p* < 0.01 versus HT29; all other variations are insignificant.

Proliferative activity and glucose consumption increased in the following order: FHC < HT29 < Colon26 (Figures 7(a) and 7(b)). The intensity of the EPR signal increased in the same order (Figure 7(c)), which indicates an increased oxidative capacity of the cancer cells compared to the normal cells. The data were verified by conventional analytical tests, demonstrating that normal colon epithelial cells have a higher reducing capacity and lower levels of superoxide and hydroperoxides, compared to the cancer colon epithelial cells (Figures 7(d)–7(f)).

**Figure 8:**
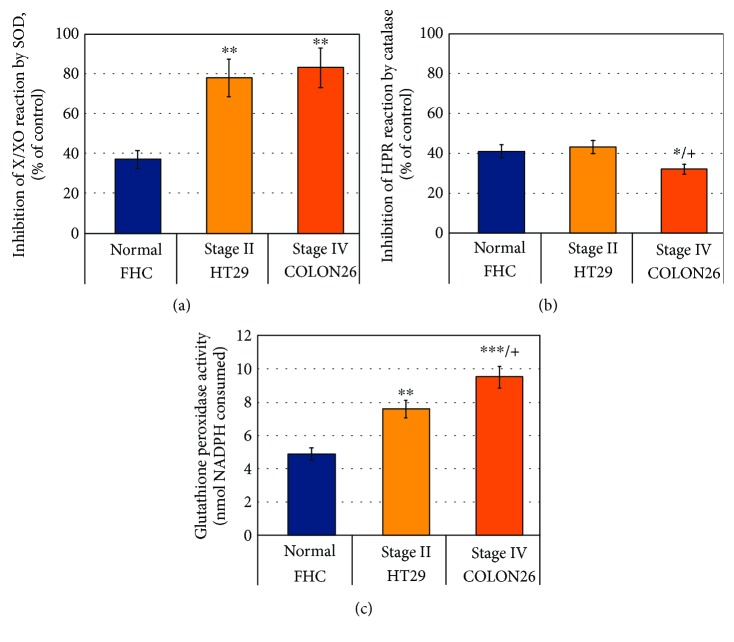
(a) Basal intracellular levels of superoxide dismutase (SOD) activity. (b) Basal intracellular levels of catalase activity. (c) Basal intracellular levels of glutathione peroxidase activity. In (a), (b), and (c), the data were obtained in cell lysates with equal protein concentration. The data are the means ± SD from three independent experiments. ^∗^ *p* < 0.05, ^∗∗^ *p* < 0.01, and ^∗∗∗^ *p* < 0.001 versus FHC; +*p* < 0.05 versus HT29; all other variations are insignificant. HRP: horseradish peroxidase; X/XO: xanthine/xanthine oxidase.

We also observed that colon cancer cells HT29 and Colon26 are characterized by higher SOD and GSH-Px activities compared to the normal colon cells FHC (Figures 8(a) and 8(c)). The catalase activity was equal in FHC and HT29, but slightly lower in Colon26 (Figure 8(b)). Nevertheless, the levels of superoxide in HT29 and Colon26 were much higher than those in FHC line (Figure 7(a)).

These data are in agreement with several experimental and clinical studies. Aykin-Burns et al. have reported that colon cancer cells (HT29, HCT116, and SW480) are characterized by a significant increase in DHE fluorescence and DCF fluorescence compared to the normal colon epithelial cells (FHC) and normal colon fibroblasts (33Co) [45]. Moreover, the levels of superoxide and hydroperoxides increase with increasing the stage of differentiation. The authors observe the same tendency between normal mammary epithelial cells (HMEC) and breast cancer cells (MB231).

Skrzydlewska et al. have reported a significant increase of lipid peroxidation products and decrease of antioxidant capacity in 81 human primary colorectal cancers, compared to the control samples, collected from macroscopically unchanged colon regions of the most distant location to the cancer [46]. The changes follow the cancer development: the highest levels of lipid peroxidation products and the lowest levels of antioxidants are observed in stage IV. The same authors report a significant increase in the activity of SOD and GSH-Px, as well as a significant decrease in the catalase activity with the tumor progression. They consider Mn-SOD/catalase and Mn-SOD/GSH-Px ratios potential biomarkers during progression from tumor growth to metastasis.

Kocot et al. have observed that SOD activity increases in the early stage of colon cancer, but markedly decreases in stage IV [47]. Miar et al. have shown that Mn-SOD/catalase and Mn-SOD/GSH-Px ratios increase in colon, lung, prostate, and liver tumors, derived from over 240 patients, which is accompanied by the increase of hydrogen peroxide [48]. The authors conclude that there is a clear redox imbalance in the early and intermediate stages of those tumors and the higher concentration of hydrogen peroxide favors proliferation, migration, and invasion in more aggressive tumors. They mentioned the relative ratio superoxide/hydrogen peroxide as a key factor. However, they did not investigate the activities of cytosolic Cu/Zn-SOD, GSH-Px, and superoxide level and did not calculate the ratio superoxide/hydrogen peroxide. Obviously, overexpression of SOD and especially Mn-SOD in aggressive tumors has a significant impact on their development and invasion. However, this does not prove the “prooncogenic” role of hydrogen peroxide. We also assume that the ratio superoxide/hydrogen peroxide could be crucial for cancer diagnostic and development of therapeutic strategy. The choice of cells at redox signaling (proliferation or apoptosis), as well as their malignancy, may depend on this ratio.

It should be noted that conventional fluorophores (such as DHE, DCF, and MitoSOX) can not evaluate the relative ratio superoxide/hydrogen peroxide in the cell suspension, because the level of superoxide can not be measured in absolute units [11]. It is impossible to calculate the molar concentration of superoxide in cell specimens, using fluorescent probes as DHE or MitoSOX, and to compare with the data for hydrogen peroxide, obtained in absolute units (moles) using DCF-based probes. In fact, DCF is not highly specific for hydrogen peroxide and its fluorescence could be affected by other hydroperoxides, as well as by reactive nitrogen species [12]. Nevertheless, it is the most used analytical test for hydrogen peroxide in cell suspensions.

It is interesting to note that nitroxides react with superoxide in a single chemical reaction [12]. This minimizes the potential artifacts arising from the multistep redox reactions characterized by “redox cycling,” which is typical for other redox-sensitive compounds (such as fluorescent and chemiluminescent) [12]. In this context, nitroxide-enhanced EPR spectroscopy offers a possibility to calculate the steady-state intracellular amount of superoxide in absolute units by using mito-TEMPO or other appropriate cyclic nitroxides as a standard. However, this assumption needs further experiments and verification.

Dual effects of superoxide and hydrogen peroxide on cell signaling regulation have been established and discussed in the literature. It seems that superoxide and hydrogen peroxide activate different signaling pathways with opposite effects on the cells—survival or apoptosis. Interesting hypothesis was described by Pervaiz and Clement [36]. The authors consider superoxide as “oncogenic ROS” and hydrogen peroxide as “oncosuppressive ROS.” They suggest that the cellular state, where the ratio tilts predominantly in favor of superoxide, inhibits apoptosis and promotes cell survival. If the ratio tilts in favor of hydrogen peroxide, this creates an intracellular environment suitable for the induction of apoptosis and cell death. It should be clarified that the authors do not deny the important role of superoxide and hydrogen peroxide in normal cellular signaling. They suggest that superoxide above a certain (threshold) level may trigger carcinogenesis. On the other hand, hydrogen peroxide above a certain (threshold) level may trigger apoptosis.

This hypothesis is well grounded by numerous experimental and empirical observations on cells, animals, and humans, which are summarized in several excellent review articles [4, 5, 36, 49, 50]. The studies report that almost all types of cancer cells are characterized by overproduction of superoxide and permanent oxidative stress due to their mitochondrial dysfunction and upregulated NOX. In addition, the proliferative and metastatic potential of cancer cells increases as the level of superoxide increases [37, 45, 51]. Many studies also show that hydrogen peroxide (in abnormal concentrations) induces apoptosis and can be considered an apoptotic factor [4, 36, 45, 50–52]. Overexpression of SOD and SOD mimetics abrogate the growth and proliferation of cancer cells in vitro and in vivo, which is indirect evidence about the “oncosuppressive” role of SOD and hydrogen peroxide [53, 54]. On the other hand, many cancer cell lines (isolated predominantly from aggressive tumors) are characterized by overexpression of SOD and especially mitochondrial Mn-SOD [47, 52–55]. Such are the cell lines from colon cancer, osteosarcoma, and glioblastoma, used in our study (Colon26, OSC19, and U87) (Figure 8(b)). We observed that the levels of hydroperoxides in these cell lines were almost equal to those in slowly proliferative noncancer cells (e.g., HaCaT and HUVEC), while the levels of superoxide were 2-5 times higher (Figures 4(d) and 4(e)). The reason might be in the different origin and nature of these cell lines. In cells of the same origin (as colon mucosa), but with different SOD activities and proliferative activities, we observed a very good correlation between the levels of superoxide and hydroperoxides—both increasing with increasing malignancy (Figures 7(e) and 7(f)). Therefore, analyzing only a single redox-active compound is not sufficient to conclude about the cellular redox status. We need to have a methodology, which gives us information about the real balance between intracellular oxidizers and reducers. Nitroxide-enhanced EPR spectroscopy is close to this goal.

Currently, we did not calculate superoxide in absolute units, using EPR spectroscopy, and can not say which of the two forms dominates. However, our data, as well as the data in the literature, suggest that aggressive tumors such as colon cancer are characterized by several distinctive features (Figure 2S-A—Supplementary Materials): (a) overproduction of superoxide that maintains mitochondrial dysfunction and genomic instability; (b) hyperactivity of SOD, especially Mn-SOD, which converts superoxide into hydrogen peroxide, trying to protect mitochondria from oxidative stress; and (c) overproduction of glutathione and hyperactivity of GSH-dependent enzymes that eliminate hydrogen peroxide and thus protect defective mitochondria from collapse [5–7, 46–48, 56]. In combination, all these events provide permanent mitochondrial dysfunction, consumption of reducing equivalents and genomic instability, strong resistance, and immortality of these cancer cells. It seems impossible to kill the aggressive cancers using standard therapeutic strategies due to this vicious cycle. We suppose that all these events ensure a permanent domination of superoxide over hydrogen peroxide in a ratio, which exceeds threshold-1 of normal cell signaling and is below threshold-2 required for the induction of apoptosis (Figure 2S-B—Supplementary Materials). The only option to kill these cancer cells is to attack all molecular targets simultaneously using combined therapy: (a) to decrease superoxide below threshold-1 and restore normal redox homeostasis or (b) to increase both types of ROS above threshold-2 and induce apoptosis. In case of nonaggressive cancers, characterized by downregulated Mn-SOD and/or downregulated GSH-dependent enzymes, it seems easier to control the situation using standard chemotherapy due to rapid induction of mitochondrial collapse through suprathreshold production of ROS (regardless of the type). However, in the aggressive tumors, this strategy is not always successful. In recent years, studies of new generation anticancer drugs have shown that overproduction of ROS is not always required to induce apoptosis in cancer cells [57].

Our data suggest that when talking about ROS-mediated anticancer effects, it is necessary to consider and specify the type of ROS. Otherwise, there is a risk of misinterpretations, because the levels of different types of ROS can change simultaneously in different directions—to increase, to decrease, or to be at a constant level during cancer progression and/or therapy. In this context, the nitroxide-enhanced EPR imaging is a useful tool to analyze the redox balance in living cells, as well as to distinguish superoxide from other reactive oxygen species. This methodology has a potential to measure superoxide in absolute units. Thus, in combination with conventional analytical tests, it will be possible in the future to calculate the ratio superoxide/hydrogen peroxide in biological objects. In addition, nitroxide probes allow redox imaging in vivo using MRI, which is a step forward in the development of a “perfect” redox sensor for the detection of tissue redox status and superoxide mapping in living organisms. At present, the efforts to design nitroxide probes for EPR/MRI imaging are focused on higher contrast, higher resistance to reduction or higher sensitivity to oxidation, and facilitated intracellular delivery, as well as BBB penetration. Significant progress has been made in this area over the past 5 years [58–62].

## 4. Conclusions

Cell-penetrating nitroxide radicals are suitable contrast probes to distinguish between nonproliferative, slow proliferative, and fast proliferative cells, by using EPR spectroscopy. The nitroxide redox cycle allows the assessment of the intracellular redox status and detection of overproduction of superoxide, which correlates with proliferative activity. The described EPR methodology is applicable on isolated cells, tissue specimens (including biopsies), and body fluids. Nitroxide-enhanced EPR spectroscopy provides greater opportunities than conventional analytical tests for the assessment of intracellular reducers and oxidizers, because the method gives information on their balance. Our study suggests that cell-penetrating nitroxide radicals are close to the “perfect” redox sensors.

## Data Availability

All data used to support the findings of this study are included within the article, as well as in the supplementary information file(s). Requests for access to the raw data should be made to Dr. Rumiana Bakalova: Quantum-State Controlled MRI Group, Institute of Quantum Life Science (QST).

## Supplementary Materials

Supplementary Materials Table 1S: redox-sensitive contrast probes and methods for detection in biological objects—merits and demerits (published data). Figure 1S: redox cycle of nitroxide and dynamics of its MRI/EPR contrast in living cells and tissues—original scheme, according to Zhelev et al. (European Journal of Cancer, vol. 49, no. 6, pp. 1467-1478, 2013). The scheme is based on Refs. [12, 17, 21–24] from the main text of the article. Figure 2S: (A) superoxide versus hydrogen peroxide in aggressive tumours—vicious cycle and therapeutic targets (adapted to Bakalova et al.; please see Ref. [56] from the main text of the article); (B) superoxide and hydrogen peroxide in cancer cell signaling, survival, and apoptosis—potential mechanisms. The scheme is based on our data and on Refs. [5–7, 46–48, 56] and from the main text of the article. Figure 3S: dynamics of EPR signal intensity of hydroxy-TEMPO (TEMPOL; 1 mM) in the presence of ascorbate (ASC; 1 : 1, mol : mol) and subsequent addition of KO_2_ (2 mM) or H_2_O_2_ (2 mM). Control—TEMPOL (1 mM) in buffer. The data on the graphic are the mean ± SD from six independent experiments. The same data were obtained with mito-TEMPO instead of TEMPOL. Figure 4S: dynamics of the EPR signal intensity of hydroxy-TEMPO (TEMPOL; 1 mM) in the presence of H_2_O_2_ (4 mM). Control—TEMPOL (1 mM) in buffer. The mean ± SD from three independent experiments is shown in (B). The same data were obtained with a higher concentration of H_2_O_2_ (up to 100 mM), as well as with mito-TEMPO instead of TEMPOL. Figure 5S: dynamics of the EPR signal intensity of mito-TEMPOH (1 mM) in the absence and presence of KO_2_ (0.5 mM). Figure 6S: dynamics of the EPR signal of mito-TEMPO (A) and mito-TEMPOH (B) in the presence of xanthine/xanthine oxidase—kinetic curves: in blue, –0.05 mM mito-TEMPO (or mito-TEMPOH), 0.5 mM xanthine, and 0.05 U/mL xanthine oxidase; in red, –0.1 mM mito-TEMPO (or mito-TEMPOH), 0.5 mM xanthine, and 0.1 U/mL xanthine oxidase. The data are the mean ± SD from five independent experiments.
