# Supplementary material for: “Redox Imaging” to Distinguish Cells with Different Proliferative Indexes: Superoxide, Hydroperoxides, and Their Ratio as Potential Biomarkers
Source: Oxid Med Cell Longev. 2019 Apr 8;2019:6373685. doi: 10.1155/2019/6373685 (PMC6476105; doi:10.1155/2019/6373685)
Supplement: Supplementary Materials — Table 1S: redox-sensitive contrast probes and methods for detection in biological objects—merits and demerits (published data). Figure 1S: redox cycle of nitroxide and dynamics of its MRI/EPR contrast in living cells and tissues—original scheme, according to Zhelev et al. (European Journal of Cancer, vol. 49, no. 6, pp. 1467-1478, 2013). The scheme is based on Refs. [12, 17, 21–24] from the main text of the article. Figure 2S: (A) superoxide versus hydrogen peroxide in aggressive tumours—vicious cycle and therapeutic targets (adapted to Bakalova et al.; please see Ref. [56] from the main text of the article); (B) superoxide and hydrogen peroxide in cancer cell signaling, survival, and apoptosis—potential mechanisms. The scheme is based on our data and on Refs. [5–7, 46–48, 56] and from the main text of the article. Figure 3S: dynamics of EPR signal intensity of hydroxy-TEMPO (TEMPOL; 1 mM) in the presence of ascorbate (ASC; 1 : 1, mol : mol) and subsequent addition of KO2 (2 mM) or H2O2 (2 mM). Control—TEMPOL (1 mM) in buffer. The data on the graphic are the mean ± SD from six independent experiments. The same data were obtained with mito-TEMPO instead of TEMPOL. Figure 4S: dynamics of the EPR signal intensity of hydroxy-TEMPO (TEMPOL; 1 mM) in the presence of H2O2 (4 mM). Control—TEMPOL (1 mM) in buffer. The mean ± SD from three independent experiments is shown in (B). The same data were obtained with a higher concentration of H2O2 (up to 100 mM), as well as with mito-TEMPO instead of TEMPOL. Figure 5S: dynamics of the EPR signal intensity of mito-TEMPOH (1 mM) in the absence and presence of KO2 (0.5 mM). Figure 6S: dynamics of the EPR signal of mito-TEMPO (A) and mito-TEMPOH (B) in the presence of xanthine/xanthine oxidase—kinetic curves: in blue, –0.05 mM mito-TEMPO (or mito-TEMPOH), 0.5 mM xanthine, and 0.05 U/mL xanthine oxidase; in red, –0.1 mM mito-TEMPO (or mito-TEMPOH), 0.5 mM xanthine, and 0.1 U/mL xanthine oxidase. The data are the mean ± SD from five ind [file 6373685.f1.doc]

**“Redox-imaging” to Distinguish Cells with Different Proliferative Index – Superoxide, Hydrogen Peroxide, and Their Ratio as Potential Biomarkers**

Zhivko Zhelev^1,2^, Ekaterina Georgieva^1^, Dessislava Lazarova^3^, Severina Semkova^2,4^,Ichio Aoki^4,5^, Maya Gulubova^1^, Tatsuya Higashi^4^, Rumiana Bakalova^3,4,5*^

^1^Medical Faculty, Trakia University, 11 Armejska Str., Stara Zagora 6000, Bulgaria

^2^Institute of Biophysics and Biomedical Engineering, Bulgarian Academy of Sciences, 21 Acad. G. Bonchev Str., Sofia 1113, Bulgaria

^3^Medical Faculty, Sofia University, 1 Koziak Str., Sofia 1407, Bulgaria

^4^Department of Molecular Imaging and Theranostics, and ^5^Group of Quantum-state Controlled MRI, National Institute of Radiological Sciences (QST-NIRS), 4-9-1 Anagawa, Chiba 263-8555, Japan

**Supplementary materials**

**Table 1S.** Redox-sensitive contrast probes and methods for detection in biological objects – merits and demerits (published data).

| **Contrast probe** | **Specificity to redox-active form** | **Basic methods for detection** | **Demerits** | **Ref.** |
| --- | --- | --- | --- | --- |
| Chemiluminescent probes:   - Lucigenine - Luminol - MCLA^1^ - Coelenterazine | O_2_•- and other ROS  Intracellular & Extracellular | Chemiluminescence  (in vitro & in vivo) | Non-specific  Redox-cycling^2^ | [1, 2] |
| - Dihydroethidium (DHE) | O_2_•- specific  Intracellular (mainly cytoplasmic) & Extracellular | Fluorescence:  FS,^3^ FC,^4^ FM^5^ (cells; difficult) | Qualitative, but not quantitative analysis  In vitro only | [3-6] |
| - MitoSOX | O_2_•- specific  Mainly mitochondrial | Fluorescence:  FS,^3^ FC,^4^ FM^5^ (cells) | Qualitative, but not quantitative analysis  In vitro only | [6-9] |
| Dichlorodihydrofluorescein (DCF)-based probes:   - DCFH-DA - DCFH-DiOxyQ | H_2_O_2_ and other peroxides specific  Intracellular (mainly cytosolic) | Fluorescence:  FS,^3^ FC,^4^ FM^5^ (cells) | Non-direct detection of H_2_O_2_  Interaction with •OH, •NO_2_, HOCl, ONOO^-^, Me^+^,^6^ cyt. c, etc.  In vitro only | [10-12] |
| - Dihydrorhodamine (DHR) | ONOO• specific | Fluorescence:  FS,^3^ FC,^4^ FM^5^ (cells) | Interaction of DHR-radical intermediate with thiols and ascorbate  In vitro only | [5, 13] |
| - Amplex Red | H_2_O_2_ specific  Extracellular only or  from isolated mitochondria | Fluorescence:  FS,^3^ FC,^4^ FM^5^ (cells) | Redox-cycling^2^  Autooxidation  Non-applicable at high concentrations of O_2_•-  In vitro only | [14-17] |
| - Cytochrome c (cyt. c) | O_2_•- specific  Extracellular | Spectrophotometric | Cyt. c can be directly reduced from electrons of other molecules  In vitro only | [18] |
| Fluorescent protein-based probes:^7^   - HyPer - pHyPer-dMito - roGFP2 - roGFP2-Orp1 - Grx1-roGFP2 - Grx1-roGFP1 - Grx1-xYFP | H_2_O_2_ specific  Intracellular  The probes could be specific to particular organel  GSH specific  Intracellular | Fluorescence:  FS,^3^ FC,^4^ FM^5^ (cells)  Fluorescence:  FS,^3^ FC,^4^ FM^5^ | Expensive  Can affect the normal redox-homeostasis  In vitro only  In vitro  In vivo (on transgenic mice only) | [19-22]  [23-26] |
| FRET-based proteins:^7, 8^   - QNO | NO specific | Fluorescence:  FM^5^ | Expensive  In vitro  In vivo (limited) | [27, 28] |
| Boronate-based fluorescent probes:   - Peroxyresorufin-1 (red) - Peroxyfluor-1 (green) - Peroxyxantone-1 (blue) - MitoPY1 (yellow) - CBA^9^ | H_2_O_2_ most specific  Intracellular  Mitochondrial H_2_O_2_ specific  ONOO• specific | Fluorescence:  FS,^3^ FC,^4^ FM^5^ (cells) | In vitro only | [29-32] |
| Cell Phasor approach | NAD(P)H specific | Fluorescence  In vitro & In vivo |  | [33-35] |
| - Ligand-conjugated microbubbles^10^ - Gas-forming molecules^11^ - Micromotor converters (MMCs)^12^ | ROS & Oxidative stress locuses | Contrast-enhanced ultrasonography  In vivo | Non-specific | [36, 37] |
| Glucose-sensitive radiotracers:   - [^18^F]-FDG^13^ | Indirect detection of ROS via glucose consumption | PET/SPECT  In vitro & In vivo | Indirect  No specificity to particular ROS | [38, 39] |
| Thiol-sensitive radiotracers:   - [^99m^Tc]-HMPAO^14^ - [^99m^Tc]-MIBI^15^ - etc. | Thiol specific  Oxidative stress locuses | SPECT  In vitro & In vivo | Indirect | [40, 41] |
| pO_2_-sensitive radiotracers:   - [^18^F]-FMISO^16^ - [^18^F]-, [^124^I]-, [^123^I]-azomycin derivative - [^62^Cu]-, [^64^Cu]-PTSM^17^ - [^99m^Tc]-ATSM^18^ - etc. | pO_2_ specific  Hypoxia locuses | PET/SPECT  In vitro & In vivo | Indirect | [42-45] |
| Antioxidant-sensitive radiotracers:   - [^99m^Tc]-GSH - [^11^C]-idebenone - [^11^C]-Coenzyme Q - [^18^F]F-BCPP-EF^19^ - etc. | Oxidative stress locuses  Mitochondrial activity detection | PET/SPECT  In vitro & In vivo | Indirect | [46, 47]  [48, 49] |

*^1^MCLA: 6-(4-Methoxyphenyl)-2-methyl-dihydroimidazo[1,2-a]pyrazin-3-one; ^2^Redox-cyclig: The radical, derived from the probe, interacts with O_2_ to generate O_2_•-. ^3^FS: Fluorescence spectroscopy; ^4^FC: Flow cytometry; ^5^FM: Fluorescence microscopy; ^6^Me^+^: Transizion metal ions. ^7^These probes are genetically coded. ^8^FRET: Fluorescence resonance energy transfer. ^9^CBA: Coumarin-7-boronic acid. ^10^Lipid-shelled decafluorobutane microbubbles, conjugated with ligands for endothelial cell adhesion molecules; ^11^Allylhydrazine and allylhydrasine in liposomes; in the presence of ROS, both substances are oxidized to gas products (nitrogen and propylene). ^12^MMCs produce microbubbles in the presence of H_2_O_2_. ^13^FDG: fluorodeoxyglucose. ^14^HMPAO: Hexamethylpropyleneamine oxime. ^15^MIBI: Methoxyisobutylisonitrile. ^16^FMISO: Fluoromisonidazole. ^17^PTSM: Pyruvaldehyde-bis(N4-methyl-thiosemicarbazone). ^18^ATSM: Diacetyl-bis(N4-methylthiosemicarbazone). ^19^BCPP-EF: 2-tert-butyl-4-chloro-5{6-[2-(2-fluoroethoxy)-ethoxy]-pyridin-3-ylmethoxy}-2H-pyridazin-3-one.*

**References**

1. J. Vasquez-Vivar, N. Hogg, K.A. Jr. Pritchard, P. Martasek, and B. Kalyanaraman, “Superoxide anion formation from lucigenin: an electron spin resonance spin-trapping study,” *FEBS Letters,* 1997, vol. 403, no. 2, pp. 127-130, 1997.
2. R. Creton, and L.F. Jaffe, “Chemiluminescence microscopy as a tool in biomedical research,” *Biotechniques,* vol. 31, no. 5, pp. 1098-1100, 2001.
3. L. Benov, L. Sztejnberg, and I. Fridovich, “Critical evaluation of the use of hydroethidine as a measure of superoxide anion radical,” *Free Radical Biology and Medicine,* vol. 25, no. 7, pp. 826-831, 1998.
4. J. Zielonka, and B. Kalyanaraman, “ROS-generating mitochondrial DNA mutations can regulate tumor cell metastasis – a critical commentary,” *Free Radical Biology and Medicine,* vol. 45, no. 9, pp. 1217-1219, 2008.
5. B. Kalyanaraman, V. Darley-Usmar, K.J. Davies, P.A. Dennery, H.J. Forman, M.B. Grisham, G.E. Mann, K. Moore, L.J. Roberts, and H. Ischiropoulos, “Measuring reactive oxygen and nitrogen species with fluorescent probes: challenges and limitations,” *Free Radical Biology and Medicine,* vol. 52, no. 1, pp. 1-6, 2012.
6. H. Zhao, S. Kalivendi, H. Zhang, J. Joseph, K. Nithipatikom, J. Vasquez-Vivar, and B. Kalyanaraman, “Superoxide reacts with hydroethidine but forms a fluorescent product that is distinctly different from ethidium: potential implications in intracellular fluorescence detection of superoxide,” *Free Radical Biology and Medicine,* vol. 34, no. 11, pp. 1359-1368, 2003.
7. K.M. Robinson, M.S. Janes, M. Pehar, J.S. Monette, M.F. Ross, T.M. Hagen, M.P. Murphy, and J.S. Beckman, “Selective fluorescent imaging of superoxide in vivo using ethidium-based probes,” *Proceedings of the National Academy of Sciences of the USA,* vol. 103, no. 41, pp. 15038-15043, 2006.
8. J. Zielonka, J. Vasquez-Vivar, and B. Kalyanaraman, “Detection of 2-hydroxyethidium in cellular systems: a unique marker product of superoxide and hydroethidine,” *Nature Protocols,* vol. 3, no. 1, pp. 8-21, 2008.
9. A.E. Dikalova, A.T. Bikineyeva, K. Budzyn, R.R. Nazarewicz, L. McCann, W. Lewis, D.G. Harrison, and S.I. Dikalov, “Therapeutic targeting of mitochondrial superoxide in hypertension,” *Circulation Research,* vol. 107, no. 1, pp. 106-116, 2010.
10. S.L. Hempel, G.R. Buettner, Y.Q. O’Malley, D.A. Wessels, and D.M. Flaherty, “Dihydrofluorescein diacetate is superior for detecting intracellular oxidants: comparison with 2`,7`-dichlorodihydrofluorescein diacetate, 5(and 6)-carboxy-2`,7`-dichlorodihydrofluorescein diacetate, and dihydrorhodamine 123,” *Free Radical Biology and Medicine,* vol. 27, no. 1-2, pp. 146-159, 1999.
11. M. Karlsson, T. Kurz, U.T. Brunk, S.E. Nilsson, and C.I. Frennesson, “What does the commonly used DCF test for oxidative stress really show?” *Biochemical Journal,* vol. 428, no. 2, pp. 183-190, 2010.
12. M.G. Bonini, C. Rota, A. Tomasi, and R.P. Mason, “The oxidation of 2′,7′-dichlorofluorescein to reactive oxygen species: a self-fulfilling prophesy?” *Free Radical Biology and Medicine,* vol. 40, no. 6, pp. 968-975, 2006.
13. B. Kalyanaraman, “Oxidative chemistry of fluorescent dyes: implications in the detection of reactive oxygen and nitrogen species,” *Biochemical Society Transactions,* vol. 39, no. 5, pp. 1221-1225, 2011.
14. B. Zhao, K. Ranguelova, J. Jiang, and R.P. Mason, “Studies on the photosensitized reduction of resorufin and implications for the detection of oxidative stress with Amplex Red,” *Free Radical Biology and Medicine,* vol. 51, no. 1, pp. 153-159, 2011.
15. M. Zhou, Z. Diwu, N. Panchuk-Voloshina, and R.P. Haugland, “A stable nonfluorescent derivative of resorufin for the fluorometric determination of trace hydrogen peroxide: applications in detecting the activity of phagocyte NADPH oxidase and other oxidases,” *Analytical Biochemistry,* vol. 253, no. 2, pp. 162-168, 1997.
16. J. Zielonka, M. Zielonka, A. Sikora, J. Adamus, J. Joseph, M. Hardy, O. Ouari, B.P. Dranka, and B. Kalyanaraman, “Global profiling of reactive oxygen and nitrogen species in biological systems: high-throughput real-time analyses,” *Journal of Biological Chemistry,* vol. 287, pp. 2984-2995, 2012.
17. V. Mishin, J.P. Gray, D.E. Heck, D.L. Laskin, and J.D. Laskin, “Application of the Amplex red/horseradish peroxidase assay to measure hydrogen peroxide generation by recombinant microsomal enzymes,” *Free Radical Biology and Medicine,* vol. 48, no. 11, pp. 1485-1491, 2010.
18. S.I. Dikalov, and D.G. Harrison, “Methods for detection of mitochondrial and cellular reactive oxygen species,” *Antioxidants and Redox Signalling,* vol. 20, no. 2, pp. 372-372, 2014.
19. V.V. Belousov, A.F. Fradkov, K.A. Lukyanov, D.B. Staroverov, K.S. Shakhbazov, A.V. Terskikh, and S. Lukyanov, “Genetically encoded fluorescent indicator for intracellular hydrogen peroxide,” *Nature Methods,* vol. 3, no. 4, pp. 281-286, 2006.
20. M. Gutscher, M.C. Sobotta, G.H. Wabnitz, S. Ballikaya, A.J. Meyer, Y. Samstag, and T.P. Dick, “Proximity-based protein thiol oxidation by H2O2-scavenging peroxidases,” *Journal of Biological Chemistry,* vol. 284, pp. 31532-31540, 2009.
21. M. Malinouski, Y. Zhou, V.V. Belousov, D.L. Hatfield, and V.N. Gladyshev, “Hydrogen peroxide probes directed to different cellular compartments,” *PLoS One,* vol. 6, no. 1, art. e14564, 2011.
22. H. Guo, H. Aleyasin, B.C. Dickinson, R.E. Haskew-Layton, and R.R. Ratan, “Recent advances in hydrogen peroxide imaging for biological applications,” *Cell and Bioscience,* vol. 4, no. 1, art. 64, 2014.
23. G. Maulucci, V. Labate, M. Mele, E. Panieri, G. Arcovito, T. Galeotti, H. Ostergaard, J.R. Winther, M. De Spirito, and G. Pani, “High-resolution imaging of redox signaling in live cells through an oxidation-sensitive yellow fluorescent protein,” *Science Signalling,* vol. 1, art. 3, 2008.
24. G. Maulucci, G. Pani, V. Labate, M. Mele, E. Panieri, M. Papi, G. Arcovito, T. Galeotti, and M. De Spirito, “Investigation of the spatial distribution of glutathione redox-balance in live cells by using Fluorescence Ratio Imaging Microscopy,” *Biosensors Bioelectronics,* vol. 25, no. 4, pp. 682-687, 2009.
25. G. Maulucci, G. Pani, S. Fusco, M. Papi, G. Arcovito, T. Galeotti, M. Fraziano, and M. De Spirito, “Compartmentalization of the redox environment in PC-12 neuronal cells,” *European Biophysics Journal,* vol. 39, no. 6, pp. 993-999, 2010.
26. C.W. Shuttleworth, “Use of NAD(P)H and flavoprotein autofluorescence transients to probe neuron and astrocyte responses to synaptic activation,” *Neurochemistry International,* vol. 56, no. 3, pp. 379-386, 2010.
27. M. Sato, N. Hida, and Y. Umezawa, “Imaging the nanomolar range of nitric oxide with an amplifier-coupled fluorescent indicator in living cells,” *Proceedings of the National Academy of Sciences of the USA,* vol. 102, no. 41, pp. 14515-14520, 2005.
28. X. Dong, C.H. Heo, S. Chen, H.M. Kim, and Z. Liu, “Quinolinebased two-photon fluorescent probe for nitric oxide in live cells and tissues,” *Analytical Chemistry,* vol. 86, no. 1, pp. 308-311, 2014.
29. E.W. Miller, A.E. Abers, A. Pralle, E.Y. Isacoff, and C.J. Chang, “Boronate-based fluorescent probes for imaging cellular hydrogen peroxide,” *Journal of the American Chemical Society,* 2005, vol. 127, no. 47, pp. 16652-16659, 2005.
30. B.C. Dickenson, C. Huynh, and C.J. Chang, “A palette of fluorescent probes with varying emission colors for imaging hydrogen peroxide signaling in living cells,” *Journal of the American Chemical Society,* vol. 132, no. 16, pp. 5906-5915, 2010.
31. H.M. Cocheme, C. Quin, S.J. McQuaker, F. Cabreiro, A. Logan, T.A. Prime, I. Abakumova, J.V. Patel, I.M. Fearnley, A.M. James, C.M. Porteous, R.A. Smith, S. Saeed, J.E. Carre, M. Singer, D. Gems, R.C. Hartley, L. Partridge, and M.P. Murphy, “Measurement of H2O2 within living Drosophila during aging using a ratiometric mass spectrometry probe targeted to the mitochondrial matrix,” *Cell Metabolism,* vol. 13, no. 3, pp. 340-350, 2011.
32. M.C. Chang, A. Pralle, E.Y. Isacoff, and C.J. Chang, “A selective, cell-permeable optical probe for hydrogen peroxide in living cells,” *Journal of the American Chemical Society,* vol. 126, no. 47, pp. 15392-15393, 2004.
33. J. Chen, S. Zhuo, R. Chen, X. Jiang, S. Xie, and Q. Zou, “Depth-resolved spectral imaging of rabbit oesophageal tissue based on two-photon excited fluorescence and secondharmonic generation,” *New Journal of Physics,* vol. 9, no. 7, pp. 212-212, 2007.
34. C. Stringari, A. Cinquin, O. Cinquin, M.A. Digman, P.J. Donovan, and E. Gratton, “Phasor approach to fluorescence lifetime microscopy distinguishes different metabolic states of germ cells in a live tissue,” *Proceedings of the National Academy of Sciences of the USA,* vol. 108, no. 33, pp. 13582-13587, 2011.
35. C. Stringari, R.A. Edwards, K.T. Pate, M.L. Waterman, P.J. Donovan, and E. Gratton, “Metabolic trajectory of cellular differentiation in small intestine by Phasor Fluorescence Lifetime Microscopy of NADH,” *Scientific Reports,* vol. 2, art. 568, 2012.
36. E.S. Olson, J. Orozco, Z. Wu, C.D. Malone, B. Yi, W. Gao, M. Eghtedari, J. Wang, and R.F. Mattrey, “Toward in vivo detection of hydrogen peroxide with ultrasound molecular imaging,” *Biomaterials,* vol. 34, no. 35, pp. 8918-8924, 2013.
37. J.K. Perng, S. Lee, K. Kundu, C.F. Caskey, S.F. Knight, S. Satir, K.W. Ferrara, W.R. Taylor, F.L. Degertekin, D. Sorescu, and N. Murthy, “Ultrasound imaging of oxidative stress in vivo with chemically-generated gas microbubbles,” *Annals of Biomedical Engineering,* vol. 40, no. 9, pp. 2059-2068, 2012.
38. K-H., Jung, J.H. Lee, C.H. Thien Quach, J-Y. Paik, H. Oh, J.W. Park, E.J. Lee, S-H. Moon, and K-H. Lee, “Resveratrol suppresses cancer cell glucose uptake by targeting reactive oxygen species-mediated hypoxia-inducible factor-1a activation,” *Journal of Nuclear Medicine,* vol. 54, pp. 2161-2167, 2013.
39. L. Masconi, “Glucose metabolism in normal aging and Alzheimer’s disease: Methodological and physiological considerations for PET studies,” *Clinical and Translational Imaging,* vol. 1, no. 4, 2013.
40. N.A. Lassen, A.R. Andersen, L. Friberg, and O.B. Paulson, “The retention of [^99m^Tc]-d,l-HMPAO in the human brain after intracarotid bolus injection: a kinetic analysis,” *Journal of Cerebral Blood Flow and Metabolism,* vol. 8, no. 6, pp. S13-S22, 1988.
41. T. Sasaki, H. Toyama, K-I. Oda, and M. Senda, “Assessment of antioxidative ability in brain: Technetium-99m-meso-HMPAO as an imaging agent for glutathione localization,” *Journal of Nuclear Medicine,* vol. 37, no. 10, pp. 1698-1701, 1996.
42. J.R. Ballinger, “Imaging hypoxia in tumors,” *Seminars in Nuclear Medicine,* vol. 31, no. 4, pp. 321-329, 2001.
43. A. Nunn, K. Linder, and H.W. Strauss, “Nitroimidazoles and imaging hypoxia,” *European Journal of Nuclear Medicine,* vol. 22, pp. 265-280, 1995.
44. J.A. O’Donoghue, J.G. Guillem, H. Schoder, N.Y. Lee, C.R. Divgi, J.A. Ruby, J.L. Humm, S.A. Lee-Kong, E.M. Burnazi, S. Cai, S.D. Carlin, T. Leibold, P.B. Zanzonico, and C.C. Ling, “Pilot study of PET imaging of ^124^I-iodoazomycin galactopyranoside (IAZGP), a putative hypoxia imaging agent, in patients with colorectal cancer and head and neck cancer,” *EJNMMI Research,* vol. 3, no. 1, art. 42, 2013.
45. G. Maulucci, G. Bacic, L. Bridal, H. Schmidt, B. Tavitian, T. Viel, H. Utsumi, A.S. Yalcin, and M. De Spirito, “Imaging of ROS-induced modifications in living cells,” *Antioxidants and Redox Signaling,* vol. 24, no. 16, pp. 939-957, 2016.
46. A.R. Fritzberg, D.M. Lyster, and D.H. Dolphin, “^99m^Tc-glutathione: role of reducing agent on renal retention,” I*nternational Journal of Nuclear Medicine and Biology,* vol. 5, no. 2-3, pp. 87-92, 1978.
47. D-W. Kim, W.H. Kim, M.H. Kim, C.G. Kim, C-S. Oh, and J.J. Min, “Synthesis and evaluation of ^99m^Tc- DTPA-glutathione as a non-invasive tumor imaging agent in amouse colon cancer model,” *Annals of Nuclear Medicine,* vol. 28, no. 5, pp. 447-454, 2014.
48. T. Sasaki, N. Matuoka, A. Kubodera, S. Ishii, G. Goto, and M. Senda, “Synthesis of [^11^C] coenzyme Q-related compounds for in vivo estimation of mitochondrial electron transduction and redox state in brain,” *Nuclear Medicine and Biology,* vol. 26, no. 2, pp. 183-187, 1999.
49. H. Tsukada, S. Nishiyama, D. Fukumoto, M. Kanazawa, and N. Harada, “Novel PET probes ^18^F-BCPP-EF and ^18^F-BCPP-BF for mitochondrial complex I: a PET study in comparison with ^18^F-BMS-747158–02 in rat brain,” *Journal of Nuclear Medicine,* vol. 55, no. 3, pp. 473-480, 2014.


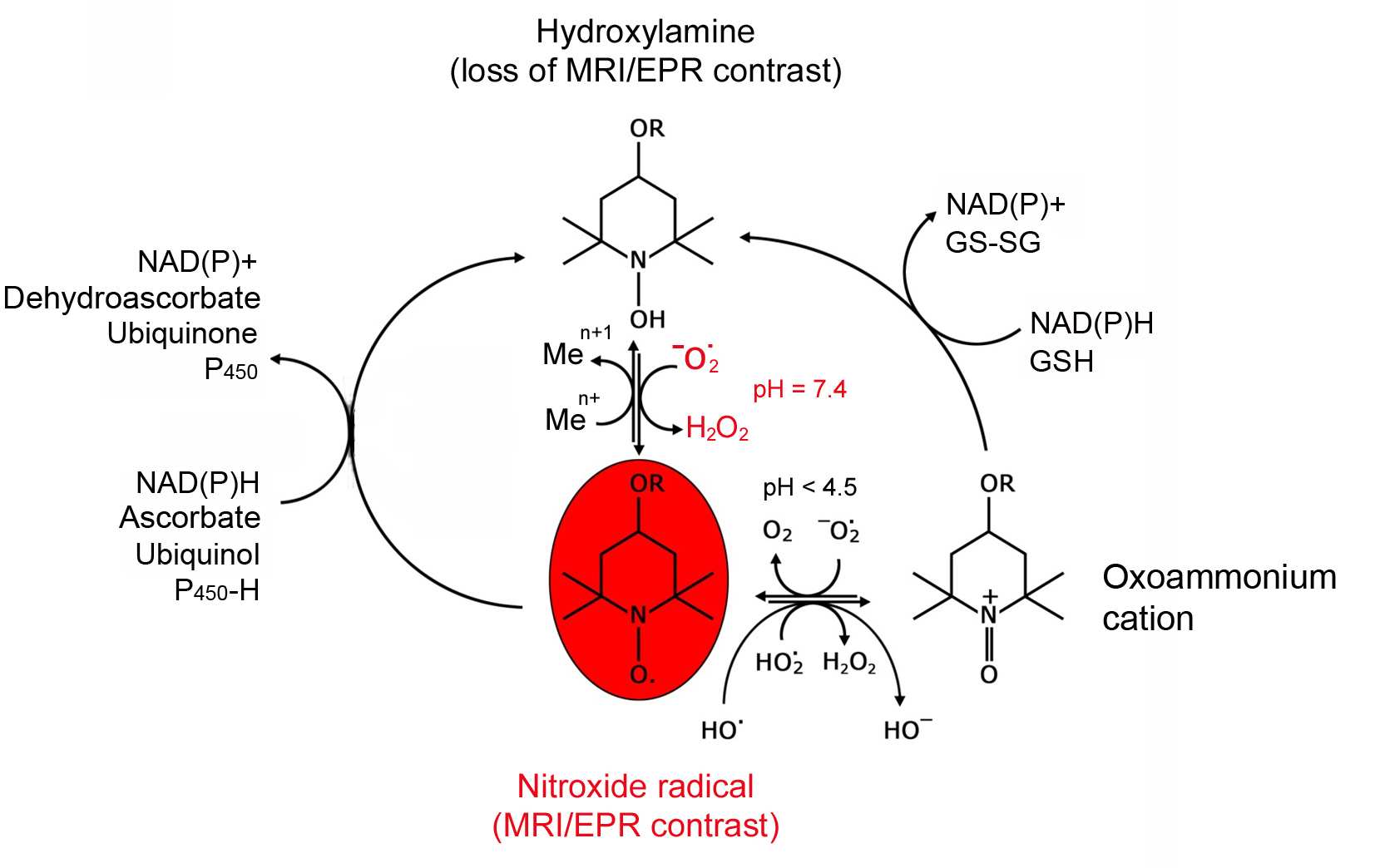


**Figure 1S.** Redox-cycle of nitroxide and dynamics of its MRI/EPR contrast in living cells and tissues – original scheme, according to Zhelev et al. (*European Journal of Cancer,* vol. 49, no. 6, pp. 1467-1478, 2013). The scheme is based on refs. 12, 17, 21-24 from the main text of the article.

**Brief description:**

It was found that nitroxide radical could be converted rapidly to the non-contrast hydroxylamine and/or oxoammonium by the following compounds: free ions of transition metals, hydroxyl and hydroperoxyl radicals, ubiquinols, NAD(P)H, ascorbate, etc. In turn, hydroxylamine and oxoammonium are superoxide dismutase (SOD) “mimetics” and could restore the nitroxide radical. The interaction of oxoammonium with superoxide occurs at pH<4.5, whereas under physiological conditions (pH~7.4) the oxoammonium is reduced by NAD(P)H to hydroxylamine. The interaction of hydroxylamine with superoxide occurs at approximately pH 7.4 with the release of hydrogen peroxide and restoration of the radical nitroxide form. It is generally accepted that in living cells and tissues, nitroxide exists primarily in two forms: as a radical and as a hydroxylamine. Various reducers and oxidizers are involved (directly or indirectly via oxoammonium) in the formation of hydroxylamine, but only the interaction of hydroxylamine with superoxide is the process that restores the nitroxide radical and its MRI/EPR contrast. Thus, the dynamics of EPR/MRI signal of cell-penetrating nitroxide radicals in cell suspensions follows the total intracellular redox-status and could serve as a marker of oxidative stress, accompanied by overproduction of superoxide.


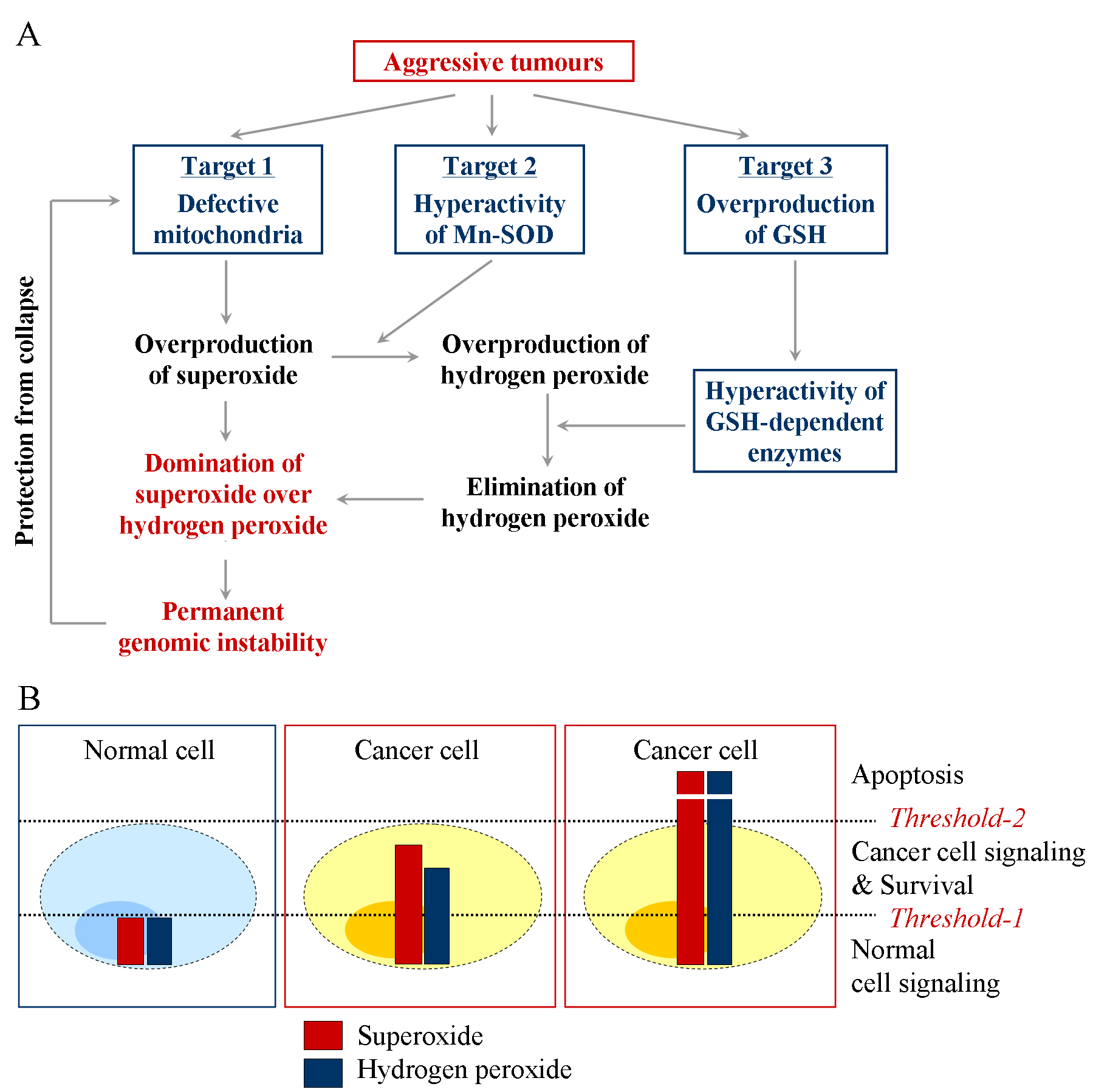


**Figure 2S.** (A) Superoxide versus hydrogen peroxide in aggressive tumours – vicious cycle and therapeutic targets (adapted to Bakalova et al.; please, see ref. 56 from the main text of the article). (B) Superoxide and hydrogen peroxide in cancer cell signalling, survival and apoptosis – potential mechanisms. The scheme is based on our data and on the refs. 5-7, 46-48, and 56 from the main text of the article.

**Brief description:**

Our data, as well as the data in the literature, suggest that aggressive tumors as colon cancer are characterized by several distinctive features (Figure 2S-A): (a) overproduction of superoxide that maintains mitochondrial dysfunction and genomic instability; (b) hyperactivity of SOD, and especially Mn-SOD, which converts superoxide into hydrogen peroxide, trying to protect mitochondria from oxidative stress; and (c) overproduction of glutathione and hyperactivity of GSH-dependent enzymes, that eliminate hydrogen peroxide and thus protect defective mitochondria from collapse. In combination, all these events provide permanent mitochondrial dysfunction, consumption of reducing equivalents and genomic instability, strong resistance and immortality of these cancer cells. It seems impossible to kill the aggressive cancers using standard therapeutic strategies due to this vicious cycle. We suppose that all these events ensure a permanent domination of superoxide over hydrogen peroxide in a ratio, which exceeds the threshold-1 of normal cell signaling and is below the threshold-2 required for induction of apoptosis (Figure 2S-B). The only option to kill these cancer cells is to attack all molecular targets simultaneously using combined therapy: (a) to decrease superoxide below the threshold-1 and restore normal redox-homeostasis; or (b) to increase both types of ROS above the threshold-2 and induce apoptosis.


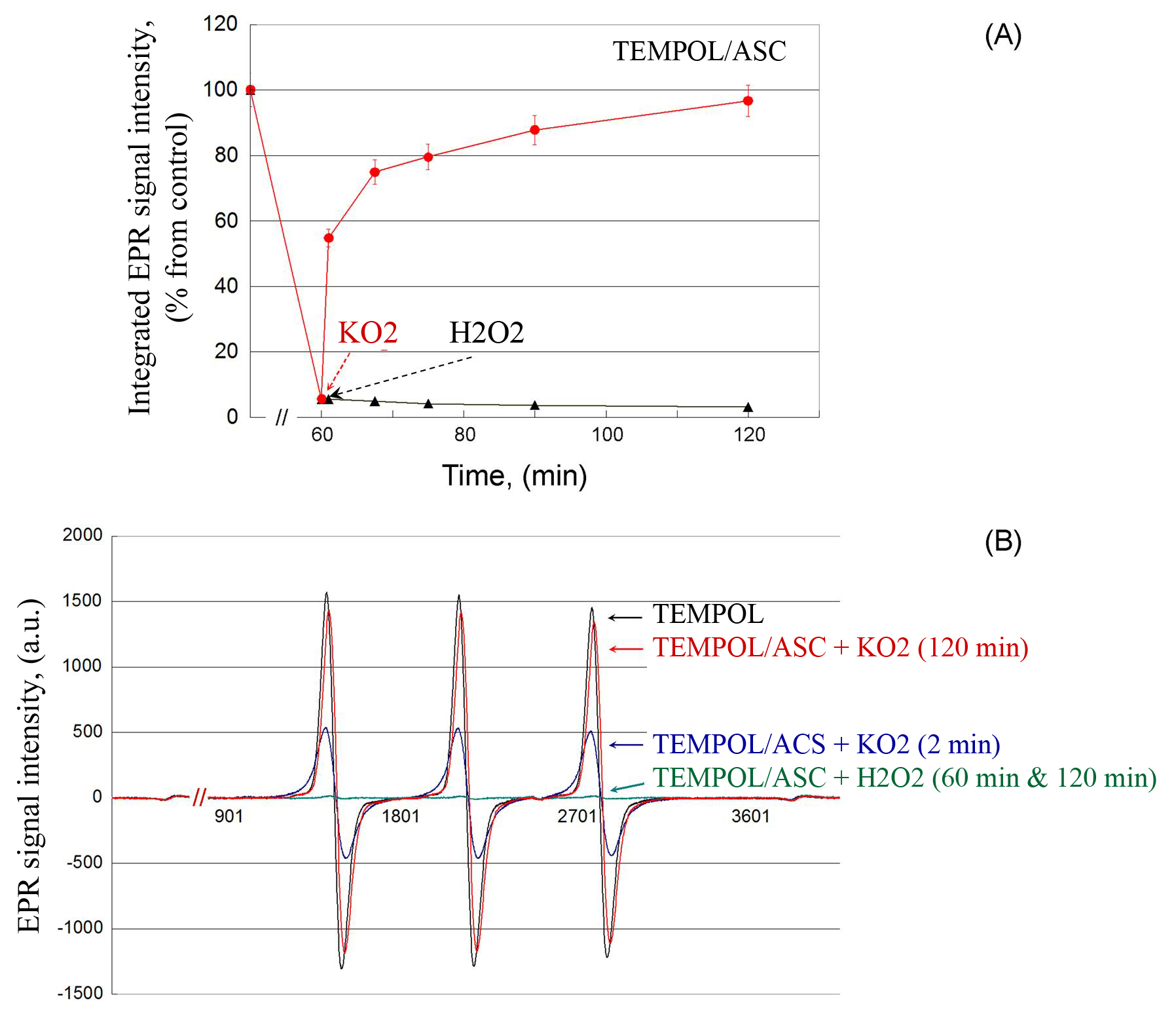


**Figure 3S.** Dynamics of EPR signal intensity of hydroxy-TEMPO (TEMPOL; 1 mM) in the presence of ascorbate (ASC; 1:1, mol:mol) and subsequent addition of КO_2_ (2 mM) or H_2_O_2_ (2 mM). Control – TEMPOL (1 mM) in buffer. The data on the graphic are mean±SD from six independent experiments. Same data were obtained with mito-TEMPO instead of TEMPOL.

**Brief description:**

TEMPOL (1 mM) was pre-incubated with ascorbate (1:1, mol:mol) within 60 min at 4 ^o^C. EPR spectrum was recorded. TEMPOL was reduced by ascorbate and EPR signal disappeared. KO_2_ (2 mМ) or H_2_O_2_ (2 mM) was added to the system “TEMPOL/Ascorbate”. EPR spectra were recorded within 1-120 min after addition of KO_2_ or H_2_O_2_. EPR signal was restored after addition of KO_2_ (red line), but not after addition of H_2_O_2_ (black line). Potassium superoxide decomposes to superoxide in water.


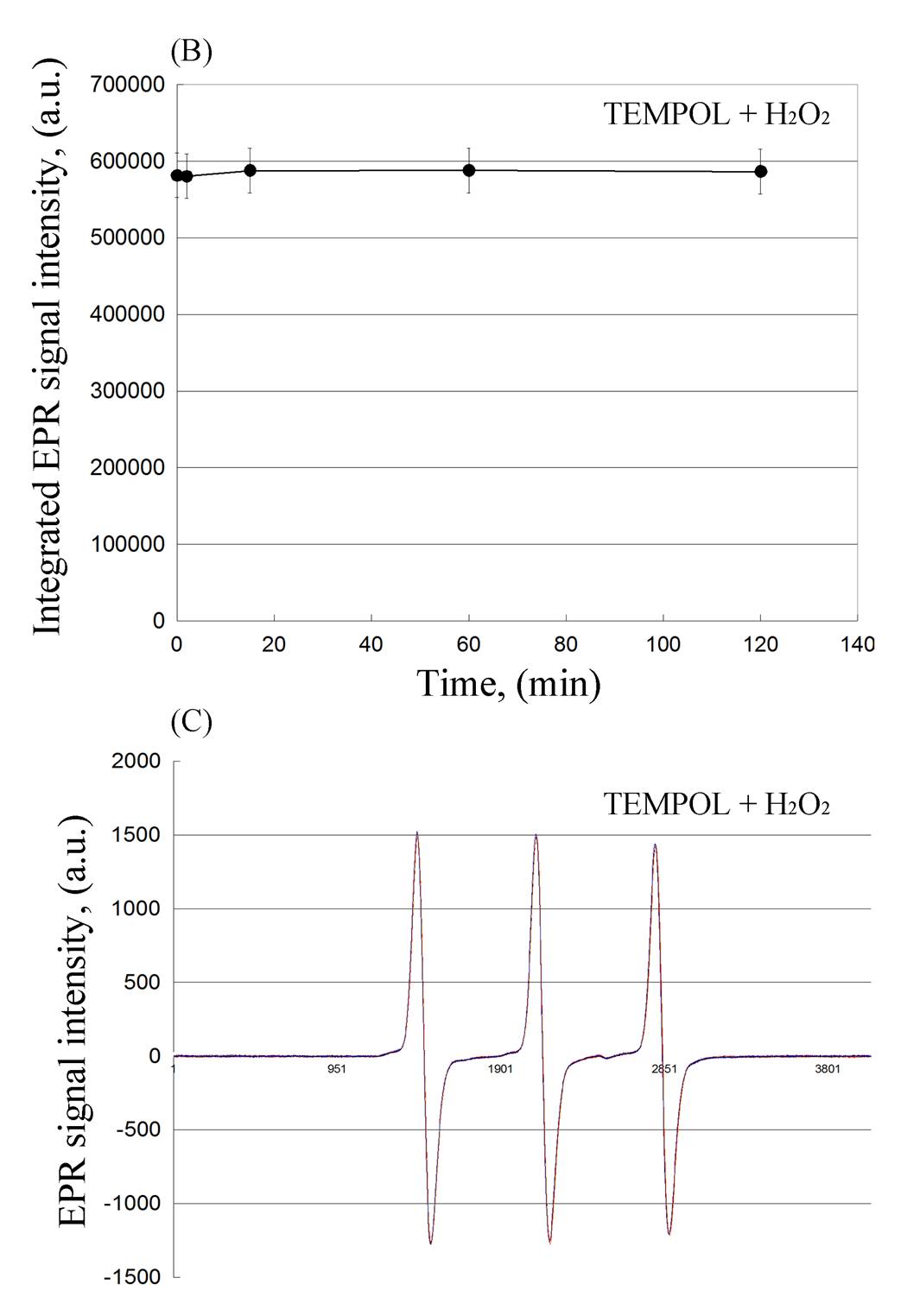


**Figure 4S.** Dynamics of EPR signal intensity of hydroxy-TEMPO (TEMPOL; 1 mM) in the presence of H_2_O_2_ (4 mM). Control – TEMPOL (1 mM) in buffer. Mean±SD from three independent experiments are shown in (B). Same data were obtained with higher concentration of H_2_O_2_ (up to 100 mM), as well as with mito-TEMPO instead of TEMPOL.


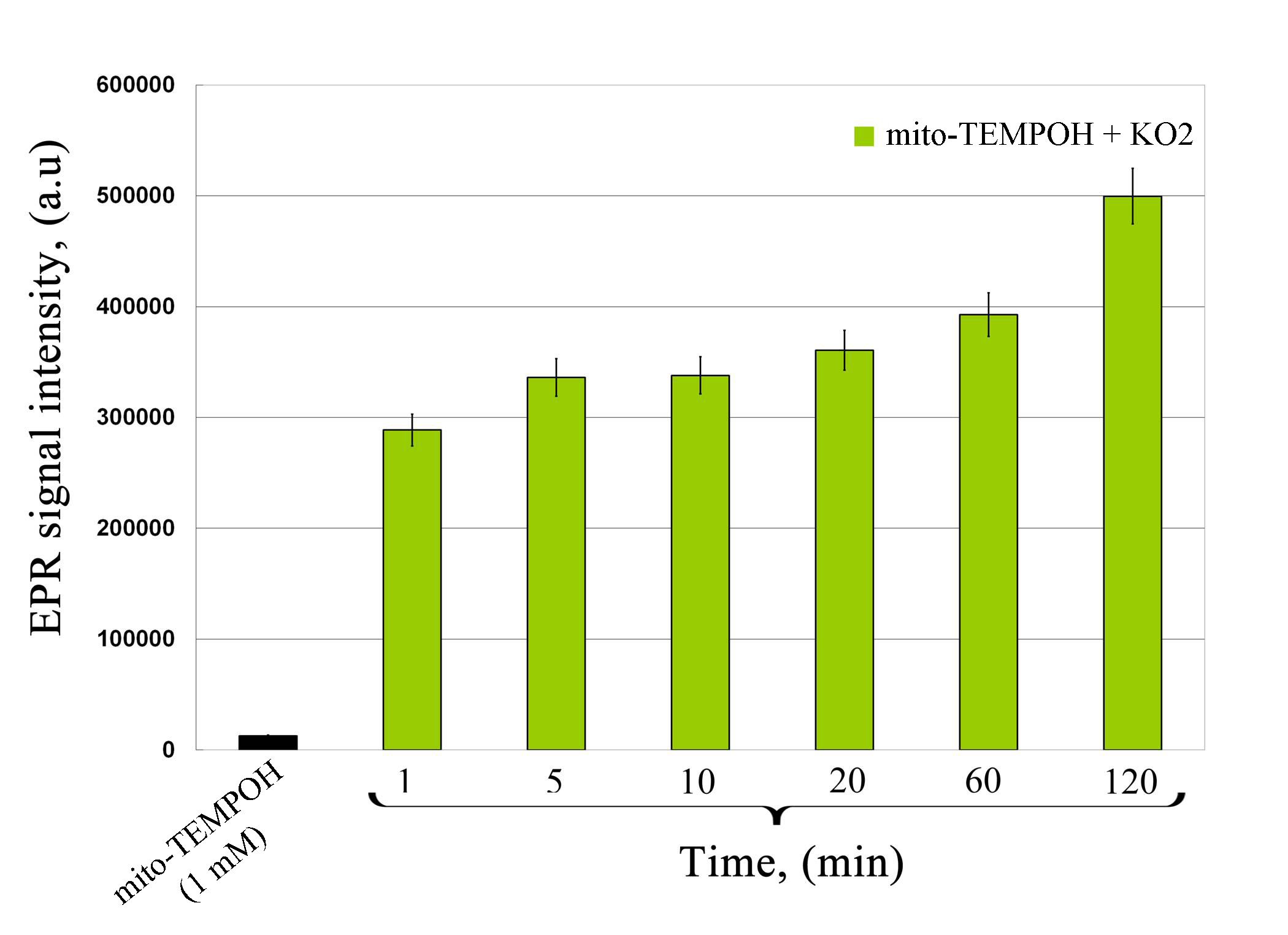


**Figure 5S.** Dynamics if EPR signal intensity of mito-TEMPOH (1 mM) in the absence and presence of КO_2_ (0.5 mM).

**Brief description:**

Mito-TEMPOH (hydroxylamine, non-contrast) is dissolved in 10 mM PBS (pH 7.4). Potassium superoxide is dissolved in DMSO (stock solution) immediately before use. EPR signal of mito-TEMPOH (very week, almost on the baseline) is recorded before and after addition of potassium superoxide at different time-intervals. Potassium superoxide decomposes to superoxide in water and EPR signal appears due to conversion of mito-TEMPOH (hydroxylamine) to mito-TEMPO (radical). The data are means±SD from three independent experiments. Hydrogen peroxide does not affect the EPR signal of mito-TEMPOH.


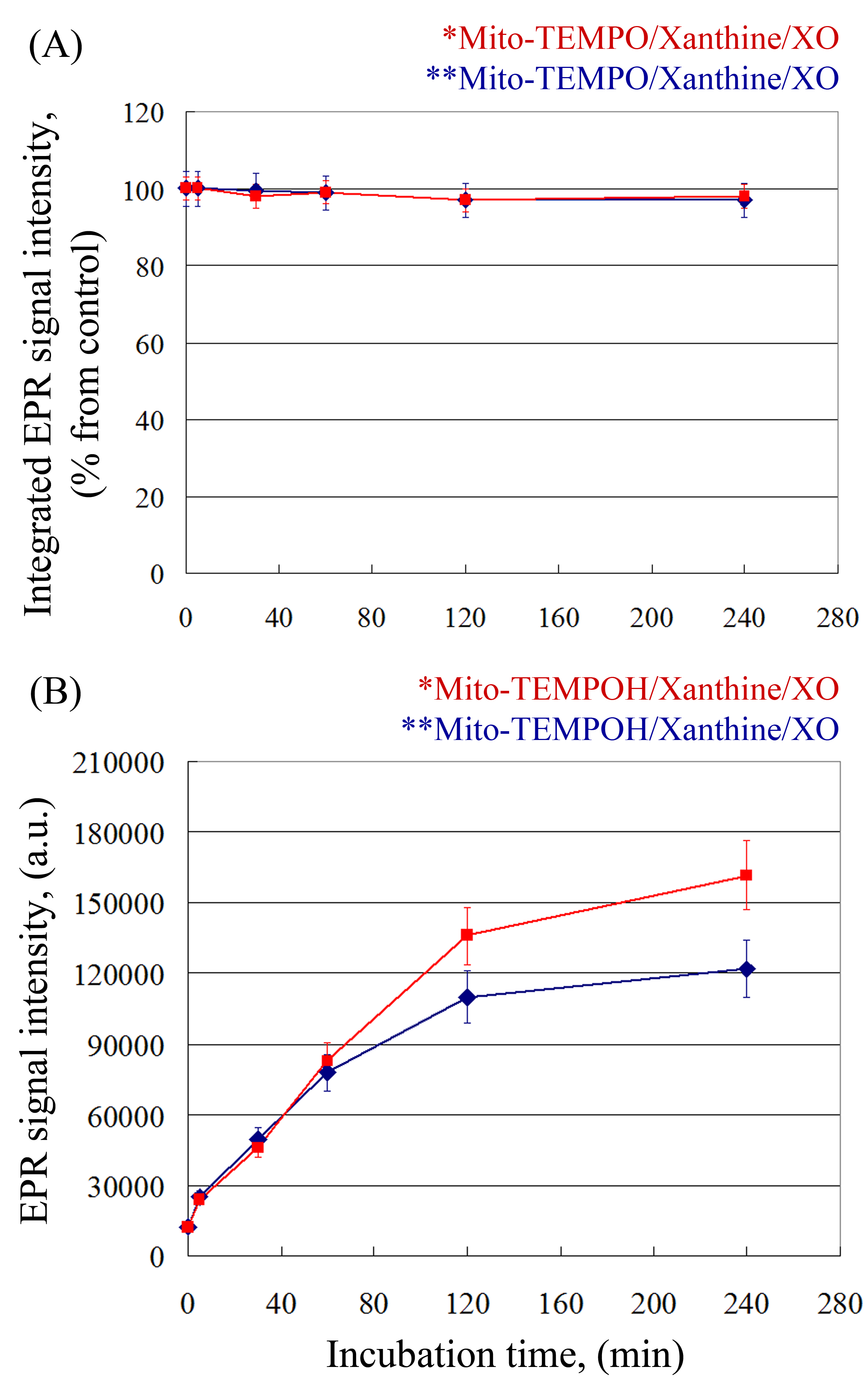


**Figure 6S.** Dynamics of EPR signal of mito-TEMPO (A) and mito-TEMPOH (B) in the presence of xanthine/xanthine oxidase – kinetic curves: In blue – 0.05 mM mito-TEMPO (or mito-TEMPOH), 0.5 mM xanthine, 0.05 U/mL xanthine oxidase; In red – 0.1 mM mito-TEMPO (or mito-TEMPOH), 0.5 mM xanthine, 0.1 U/mL xanthine oxidase. The data are Mean±SD from five independent experiments.
